# Oxovanadium(IV) Thiocarboxylate Paddlewheels Containing Ancillary Group 10 Metals: A Comparative Study on Pd and Pt Derivatives

**DOI:** 10.1021/acs.inorgchem.5c04835

**Published:** 2025-12-19

**Authors:** Olga Mironova, Giacomo Bellini, Alessio Nicolini, Manuel Imperato, Antonio Ranieri, Marco Borsari, Matteo Briganti, Rodolphe Clérac, Mathieu Rouzières, Enrico Salvadori, Maria Chiara Pagliero, Mario Chiesa, Andrea Cornia

**Affiliations:** † Dipartimento di Scienze Chimiche e Geologiche e UdR INSTM, 9306 Università degli Studi di Modena e Reggio Emilia, via G. Campi 103, Modena 41125, Italy; ‡ Dipartimento di Scienze Fisiche, Informatiche e Matematiche, Università degli Studi di Modena e Reggio Emilia, via G. Campi 213/A, Modena 41125 Italy; § Dipartimento di Scienze della Vita, 9306 Università degli Studi di Modena e Reggio Emilia, via G. Campi 103, Modena 41125, Italy; ∥ Dipartimento di Chimica “Ugo Schiff” e UdR INSTM, 9300 Università degli Studi di Firenze, via della Lastruccia 3, Sesto Fiorentino, FI 50019, Italy; ⊥ Univ. Bordeaux, CNRS, Centre de Recherche Paul Pascal, CRPP, UMR 5031, Pessac 33600, France; # Dipartimento di Chimica e NIS Centre, 9314 Università degli Studi di Torino, via P. Giuria 7, Torino 10125, Italy

## Abstract

Vanadyl-containing paddlewheel structures have recently joined the pool of molecular spin systems showing respectable coherence times (*T*_m_). We extended the investigation of [PtVO­(SOCR)_4_] (R = Me, Ph) by synthesizing the corresponding Pd derivatives. Crystals of [PdVO­(SOCMe)_4_] and [PdVO­(SOCPh)_4_]·DCM are isomorphous to their Pt analogues and comprise staggered and square dimers, respectively. For both Group 10 metals, M···M′ contacts in staggered dimers promote a stronger antiferromagnetic coupling than M···S′ contacts in square dimers, with Pd complexes showing a slightly more effective interaction consistent with DFT predictions. In solution, the paddlewheels exist in monomeric form and undergo a quasi-reversible one-electron reduction, more favorable for M = Pd than Pt and for R = Ph than Me. X-band EPR spectra in frozen CD_2_Cl_2_/toluene-*d*_8_ show the eight-line hyperfine pattern characteristic of ^51^V (*I* = 7/2), with identical spin-Hamiltonian parameters across the series. Unlike the interaction with ^195^Pt (*I* = 1/2, 34%), the superhyperfine coupling with ^105^Pd (*I* = 5/2, 22%) is unresolved. *T*_m_ values are essentially the same for Pt and Pd derivatives and follow the same substituent dependence (Ph > Me). Therefore, the ancillary Group 10 metal affects redox potentials and, to a lesser extent, solid-state magnetic properties, but leaves quantum coherence unchanged.

## Graphical abstract

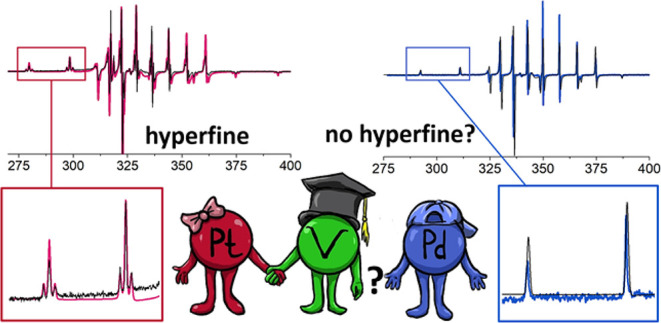

## 1 Introduction

Lantern structures, also known as paddlewheels (PWs), are robust and versatile molecular complexes widely studied in metal–organic chemistry, e.g., in catalysis and the design of metal–organic frameworks.^1–3^ Among the multitude of homo- or heterobimetallic PWs so far synthesized, examples of vanadyl (VO^2+^)-based lanterns are surprisingly scarce. All of them belong to the class of heterobimetallic PWs containing, in addition to a 3d metal (Tr), a Group 10 metal (M), specifically Pt or Pd.^4–6^ In these structures, the two metals are bridged by bidentate ligands carrying different donor atoms, like (O,S), (N,S), or (N,O). Their binding mode aligns with the concept of soft–hard Lewis acids and bases (HSAB), where hard and soft donor atoms preferentially coordinate to Tr and M, respectively.^7^

For almost a decade, Prof. Doerrer’s group has been advancing this approach and has applied it to the synthesis of heterobimetallic PWs using thioacetate and thiobenzoate (O,S) ligands,^6,8–13^ with other groups recently joining the work.^14–16^ In the course of this time, many complexes with the general formula [PtTr­(SOCR)_4_(L)_*n*_] (Tr = Cr, Mn, Fe, Ni, Co, and Zn; R = Me, Ph; L = axial ligand) have been comprehensively studied, and major regularities between their structure and magnetic properties have been established. Significantly, the Doerrer’s group was the first to isolate two VO^2+^ complexes [PtVO­(SOCR)_4_] with R = Me (**1Pt**) or R = Ph (**2Pt**), which were designed as metalloligands to enhance the performance of lanthanide complexes.^17^

We have recently highlighted the potential of these VO^2+^-based lanterns in the field of quantum information science.^18^ Vanadyl complexes are, in general, well-established molecular spin qubits with consistently long coherence times (*T*_m_). Their *S* = 1/2 electronic spin is coupled with the nuclear spin (*I* = 7/2) of ^51^V, a naturally abundant isotope (99.75%), yielding an electronuclear spin qudit.^19–21^ Among strategies to enhance their performance is reducing spin–lattice relaxation effects using rigid ligand scaffolds.^22–24^ Macrocyclic vanadyl complexes with phthalocyanines^25,26^ and porphyrins,^20,21,27^ for instance, have *T*_m_ values surpassing those of bis-ligand systems like acetylacetonates.^28–30^ Further sources of decoherence arising from neighboring electronic or nuclear spin carriers^26,31–33^ can be mitigated by diluting the qubits in a diamagnetic host (e.g., a frozen solution or a crystalline matrix^23,25^ ) and utilizing O- or S-donor ligands (*I* = 0) as well as deuterated or nuclear-spin-free solvents.^24,26,30,34^ Combining these approaches, we have recently isolated a quasi-macrocyclic bis­(β-diketonato) vanadyl complex with *T*_m_ = 13 μs in a frozen deuterated toluene-dichloromethane glass at 10 K.^35^ **1Pt** and **2Pt** also contain a V^4+^ ion coordinated solely by O donors and, in the same experimental conditions, exhibit quite respectable coherence times of 6 and 11 μs, respectively.^18^ From a design perspective, the square planar coordination geometry of the d^8^ Group 10 metal ion serves as an important structural element, imposing an idealized 4-fold symmetry to the PWs. Furthermore, the rigid structure of the M­(SOCR)_4_^2–^ metalloligands offers an alternative to organic macrocyclic systems and enables multiple routes for qubit functionalization and processing without the direct involvement of the paramagnetic center, e.g., by chemi- or physisorption on surfaces.

In this study,^36^ we continue our exploration of vanadyl PWs by focusing on Pd-based derivatives. While the influence of R substituents on the structure and properties of [PtTr­(SOCR)_4_(L)_*n*_] complexes has been extensively examined in various contexts, the impact of replacing Pt with Pd remains relatively unexplored.^15^ Our initial idea was that a lighter Group 10 metal would reduce spin–orbit coupling (SOC) effects, conferring improved quantum coherence properties on vanadyl PWs. Transitioning from Pt to Pd presents a tricky synthetic challenge due to the dual coordination nature of Pd, which can interact with both soft and hard donor atoms.^15,37–39^ By developing specialized synthetic protocols, we were able to isolate both [PdVO­(SOCMe)_4_] (**1Pd**) and [PdVO­(SOCPh)_4_] (**2Pd**), and to study their solid-state and solution behavior in direct comparison to the Pt predecessors. Importantly, these Pd PWs are isostructural to the corresponding Pt analogues and form isomorphic crystals containing dimeric assemblies supported by M···M′ or M···S′ contacts. Thus, they also provide a unique opportunity for directly investigating how the strength of magnetic coupling varies depending on the diamagnetic heavy metal ion involved. In solution, Pd derivatives exhibit distinct behavior characterized by an increased propensity for reduction. Moreover, their X-band EPR spectra in a frozen CD_2_Cl_2_/toluene-*d*_8_ matrix lack the superhyperfine line splitting introduced by the ^195^Pt isotope in **1Pt** and **2Pt**; however, their quantum coherence closely mirrors the trends observed for Pt counterparts, remaining largely unaffected by replacing Pt with the lighter Pd.

## 2 Results and Discussion

### 2.1 Synthesis

In general, for the selective synthesis of [MTr­(SOCR)_4_(L)_*n*_] complexes, the 3d metal salt is added to the corresponding metalloligand [M­(SOCR)_4_]^2–^, obtained in situ in water, which causes almost immediate precipitation of the desired product. However, the palladium analogues of **1Pt** and **2Pt** cannot be obtained by strictly following the procedures developed earlier.^15^ The reason is that the higher lability of Pd over Pt in terms of the HSAB principle favors the formation of undesired products and also speeds up the reactions. We found that the target thioacetate derivative **1Pd** cannot be obtained in water, presumably due to the prevailing formation of binary palladium­(II) sulfides.^15^ Instead, we could access **1Pd** by mixing the reagents (K_2_PdCl_4_, 4 equiv of KSOCMe, and VOSO_4_·3.6H_2_O) in anhydrous methanol under an atmosphere of purified nitrogen. The reaction mixture turned into a brown suspension after 18 h of stirring, then the solvent was evaporated, and an emerald-green product was extracted with dichloromethane (DCM). The layering of this concentrated solution with *n*-hexane afforded dark green plates of **1Pd** in 67% yield. As proof of the necessity of air-free and nonaqueous conditions, the synthesis failed when performed in degassed water under a N_2_ atmosphere or in dry methanol in the air.

In contrast to the difficulties encountered in the synthesis of **1Pd**, thiobenzoate **2Pd** was accessed by only slightly modifying the “classical” procedure used for the Pt analogue. In fact, the greater “hardness” of Pd vs Pt causes a higher propensity to form homometallic lanterns with ligands coordinated through both soft and hard donors, as found in homoleptic [Pd_2_(pyt)_4_][Bibr ref37] (Hpyt = pyridine-2-thiol) and heteroleptic [Pd_2_((C_6_H_4_)­PPh_2_)_2_(SOCPh)_2_].^38^ To reduce the formation of these homobimetallic species, a diluted solution of K_2_PdCl_4_ in water was added dropwise over 10 min to a stirred aqueous solution of NaSOCPh, resulting in a clear orangish-red solution. The addition of VOSO_4_·3.6H_2_O dissolved in water caused the formation of an ocher precipitate, which was isolated by filtration. When this precipitate was treated with DCM, a red solution flowed down, leaving a grassy-green precipitate on the filter. The green product turned out to be poorly soluble in DCM, which is usually a good solvent for this type of PWs, but could be dissolved in hot THF. Upon cooling, green crystals formed and were structurally characterized as **2Pd**·THF. In the red fraction, some green plates also formed upon standing, which were analyzed as a DCM solvate (**2Pd**·DCM). Even upon gentle drying, this solvate loses the lattice DCM completely. Larger crystals of both solvates can be grown by subjecting solutions of **2Pd** to cycles of gentle heating and slow cooling.

Solutions of **2Pd** in THF remain stable as long as they are kept under an inert atmosphere, but display signs of sensitivity to moisture upon standing in the air. For instance, a THF solution over crystals gradually turns brown in a few days, and signals attributed to diamagnetic species appear in the ^1^H NMR spectrum (see below). Presumably, this is caused by an increase in the polarity of the solvent, which facilitates structural rearrangement. However, the slow degradation rate and the nonwetting^40^ of the crude product allow for carrying out the synthesis in the air up to the recrystallization step. In contrast, solutions of **1Pd** remain visibly unchanged after weeks of storage in the air.

The IR spectra of **1Pd** and **2Pd** closely mirror those of the corresponding Pt analogues, with differences in the peak positions not exceeding 10 cm^–1^ (). In particular, the band attributed to V=O stretching shifts from 984 in **1Pt** to 995 cm^–1^ in **1Pd**, and from 989 in **2Pt** to 1002 cm^–1^ in **2Pd**.

### 2.2 X-ray Crystallography

In a square planar coordination environment, Pd­(II) and Pt­(II) have very similar ionic radii (0.64 and 0.60 Å, respectively).^41^ By consequence, compounds **1Pd** and **2Pd**·DCM are isomorphous with their Pt analogues (Figure S1). **1Pd** crystallizes in the monoclinic *C*2/c space group and the unit cell contains eight [PdVO­(SOCMe)_4_] molecules in general position. The selected geometrical parameters gathered in Table 1 show that the V–Pd distance (2.91 Å) is slightly longer than the V–Pt one in **1Pt** (2.86 Å). However, the two derivatives have virtually identical intramolecular twisting angles (τ_intra_ = 14.9 vs 15.2°).^17^ Molecules form approximately coaxial dimers (Figure 1a) with short M···M′ contacts and virtually straight V–M···M′ angles. Differences in intermolecular M···M′ and (average) M···S′ distances lie well below 0.05 Å in the two derivatives, which both have an intermolecular twisting angle of 32.6° (evaluated by averaging the smallest set of S–M···M′–S′ dihedrals). This indicates that the conformation is virtually identical in the two compounds and closer to staggered (45°) than eclipsed (0°).^6^

**Figure 1:**
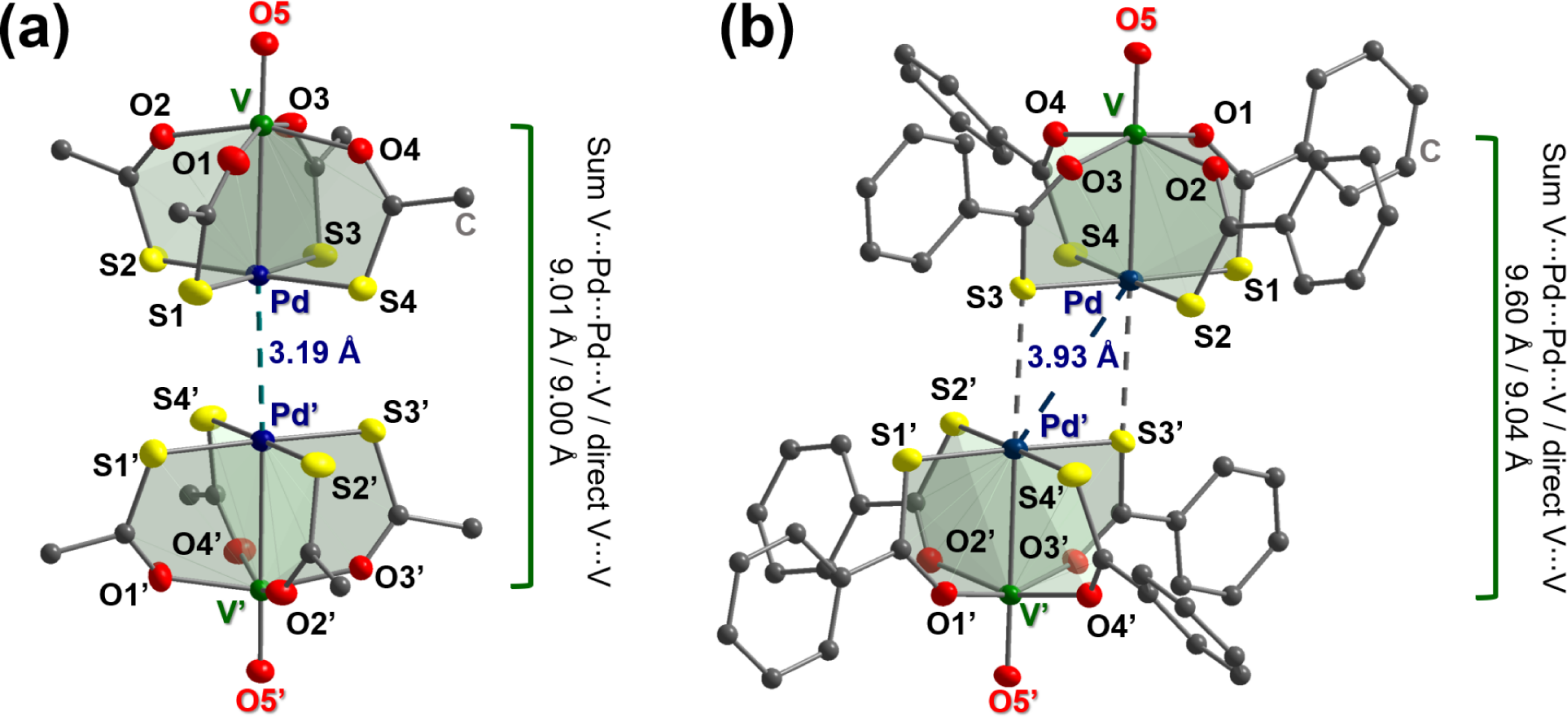
Molecular structures of dimers {**1Pd**}_2_ (staggered) and {**2Pd**}_2_ (square, in **2Pd**·DCM). Metals and heteroatoms are displayed as 50% probability ellipsoids, while carbon atoms are drawn in ball and stick style; hydrogens are omitted for clarity. Primed atoms are related to unprimed ones by 2-fold rotation in (a) and inversion in (b).

**Table 1:** Selected Bond Distances and Angles in **1Pd**, **1Pt**, **2Pd**·DCM, **2Pd**·THF, and **2Pt**·DCM^a^

	Distances (Å)			Angles (deg)	
Compound	V=O	V–M	M···M′/M···S′	V–M···M′	τ_intra_ ^b^
**1Pd**	1.5802(19)	2.9112(5)	3.1868(4)/3.97 (avg)	176.048(13)	14.9
**1Pt** ^c^	1.592(2)	2.8635(6)	3.1747(4)/3.94 (avg)	177.168(11)	15.2
**2Pd**·DCM	1.5827(13)	2.8355(3)	3.9280(3)/3.0737(5)	139.972(9)	21.3
**2Pd**·THF	1.5777(15)	2.8377(4)	4.2113(4)/3.0936(6)	134.557(10)	21.0
**2Pt**·DCM^c^	1.581(4)	2.7823(10)	3.8408(5)/3.1266(14)	143.62(2)	20.6

a Dots indicate intermolecular contacts; primed symbols are used for atoms of the neighboring PW in a dimer.

b Intramolecular twisting angle, evaluated as the average O–V–Pt–S dihedral angle (with O and S belonging to the same thiocarboxylate ligand).

c Data taken from ref 17 (structures collected at 100 K).

Both **2Pd**·DCM and **2Pd**·THF crystallize in the triclinic *P*1̅ space group and are isomorphous with each other and with **2Pt**·DCM.^17^ The unit cell contains two [PdVO­(SOCPh)_4_] molecules and two solvent molecules (DCM or THF) in general positions. The V–Pd distance (2.84 Å) is again longer than the V–Pt distance in **2Pt**·DCM (2.78 Å), although the intramolecular twisting angle is almost identical in **2Pd**·DCM/THF and **2Pt**·DCM (21.0–21.3 vs 20.6°). As in **2Pt**·DCM, the PWs assemble into noncoaxial (square) dimers (Figure 1b), with a displacement of one molecule relative to the other and formation of two reciprocating intermolecular M···S′ contacts (∼3.1 Å). By consequence, the M···M′ distances in these square dimers (3.84–4.21 Å) are considerably larger than in the staggered dimers (3.17–3.19 Å), and the displacement angles (V–M···M′) are correspondingly much more acute (134.6–143.6°). A significant difference between the three thiobenzoate derivatives is the displacement angle, which increases in the order **2Pd**·THF < **2Pd**·DCM < **2Pt**·DCM with concomitant shortening of the M···M′ separation (Table 1).

Statistics collected on the structurally characterized [MTr­(SOCR)_4_(L)_*n*_] complexes (Figure S4) show that the Tr–M distance varies within narrow limits and does not depend on the nature of the transition metal or the type of complex. On the other hand, the contact distance M···M′ is expectedly much shorter in staggered dimers than in square dimers. Predictably, a bulky axial ligand (such as quinuclidine^42^) or a large cocrystallized compound (acridine^43,44^ ) prevents the formation of dimers, but the key factors ruling the way PWs assemble into dimers have not been fully ascertained.^45^ Thioacetate PWs show a greater propensity to form staggered dimers than thiobenzoate ones, which may be caused by a higher sterical hindrance in the latter, but other factors must play a role. For instance, the three known staggered dimers with thiobenzoate ligands are found in crystals of [PtCo­(SOCPh)_4_(H_2_O)], [PtNi­(SOCPh)_4_(H_2_O)],^8^ and [PtZn­(SOCPh)_4_(H_2_O)][Bibr ref12] obtained from THF. However, the cobalt­(II) complex gives square dimers when the compound is recrystallized from DCM. By contrast, **2Pd** assembles into noncoaxial dimers upon recrystallization from both solvents.

### 2.3 Magnetic Measurements

Magnetic characterization was performed on crystalline samples of **1Pd** and **2Pd**·THF (Figure 2). For **1Pd**, the χ_M_ *T* value (χ_M_ is the magnetic susceptibility per mole of vanadyl units and *T* is temperature) is 0.37 cm^3^ K mol^–1^ at 300 K and remains approximately constant down to about 50 K. Upon further cooling, it progressively drops, reaching 0.03 cm^3^ K mol^–1^ at 1.9 K (Figure 2a). The χ_M_ *T* vs *T* data can be perfectly fitted to an *S* = 1/2 spin dimer model in the low-field approximation with *g* = 1.99(5) and *J* = 5.23(4) cm^–1^ (Table 2), which indicates antiferromagnetic interactions between vanadium­(IV) ions in neighboring complexes (*J* values are based on the *J* **S**_1_·**S**_2_ convention). Isothermal magnetization (*M*) vs applied field (*H*) data from 1.9 to 10 K exhibit a clear inflection point. They are consistent with a nonmagnetic *S* = 0 state and field-induced population of the excited *S* = 1 state, and can be fitted to give *g* = 1.97(5) and *J* = 5.16(4) cm^–1^, in perfect agreement with the analysis of χ_M_ *T* vs *T* data (Table 2). Considering that the closest V···V contact in the unit cell of **1Pd** is shorter than the intradimer V···V′ separation (Figure S2), interdimer couplings may, in principle, contribute to the observed magnetic behavior. The validity of our model is, however, supported by the quality of the fit and the intradimer *J* values estimated by DFT calculations (see below and ref 18 ).

**Figure 2:**
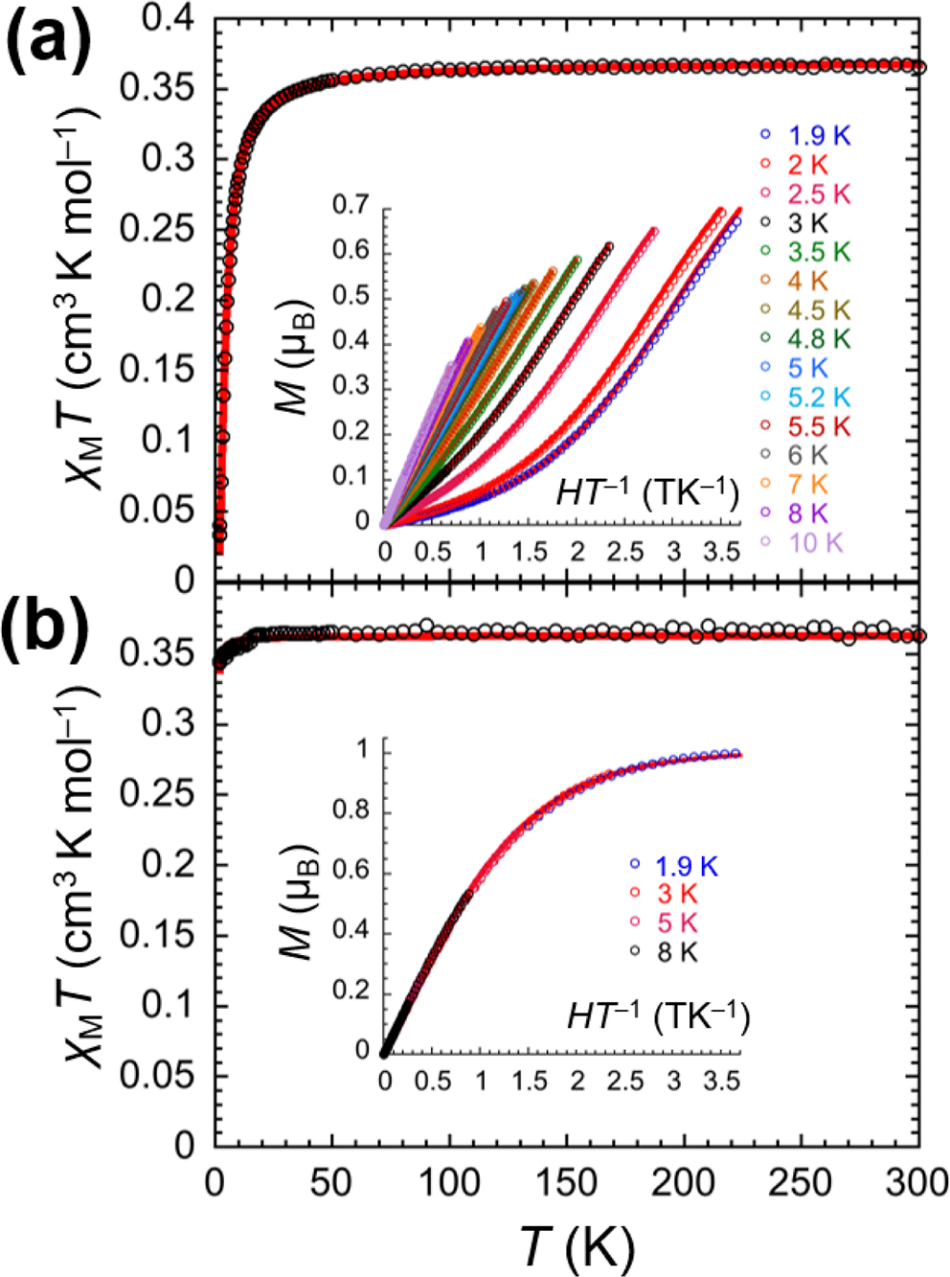
Temperature (*T*) dependence of the molar magnetic susceptibility (χ_M_) per vanadyl unit for **1Pd** (a) and **2Pd**·THF (b) measured in applied fields (*H*) of 0.1 and 1 T for temperatures below and above 15 K, respectively. Solid red lines are the best fits of the experimental data to a Heisenberg *S* = 1/2 spin dimer model. Insets: Field dependence of the magnetization (*M*) per vanadyl unit plotted as *M* vs *HT* ^–1^ for **1Pd** (a) and **2Pd**·THF (b) at the indicated temperatures. Solid red lines are the best fits of the experimental data to a Heisenberg *S* = 1/2 spin dimer model for **1Pd** (a) and a simple *S* = 1/2 Brillouin function for **2Pd**·THF (b).

**Table 2:** Superexchange Coupling Constants *J* within Dimers of **1M** and **2M** from DC Magnetic Measurements and DFT Calculations^a^

Compound	Expt.	DFT	ref.
**1Pd**	5.23(4)^b^, 5.16(4)^c^	7.44	This work
**2Pd**	0.38(1)^b^	0.79	This work
**1Pt**	4.70^b^	6.55	^17^,^18^
**2Pt**	0.644(2)^b^,^c^	0.47	^18^

a *J* uses *J* **S**_1_·**S**_2_ convention and is expressed in cm^–1^.

b From the analysis of χ_M_ *T* vs *T* data.

c From the analysis of isothermal *M* vs *H* data.

The χ_M_ *T* value for **2Pd**·THF is 0.37 cm^3^ K mol^–1^ at 300 K and undergoes only a slight decrease upon cooling (Figure 2b). χ_M_ *T* vs *T* data indeed provide *g* = 1.99(5) and *J* = 0.38(1) cm^–1^, a value indicative of a very weak antiferromagnetic interaction (Table 2). Consistent with this, the isothermal *M* vs *H* plots from 1.9 to 8 K conform to simple Brillouin behavior and can be fitted with *g* = 2.01(5) (Figure 2b).

The described dc magnetic analysis indicates that the magnetic coupling is much larger in the staggered thioacetate dimers than in the square thiobenzoate ones, in agreement with previous findings on the Pt derivatives.^17,18^ In the staggered dimers (**1M**), the magnetic coupling is mediated by the metallophilic M···M′ interaction^16^ and occurs between vanadium­(IV) ions that are 8.9–9.0 Å apart from each other. The coupling is, however, weaker than found in staggered dimers containing Co^2+^ (22–25 cm^–1^) and Ni^2+^ ions (25–120 cm^–1^).^8,10,16^ The difference is clearly rooted in the fact that the unpaired electron in vanadyl complexes is mainly localized in the 3d_*xy*_ metal orbital.^30^ The vanadium 3d_*xy*_ and palladium 4d_*xy*_ orbitals are in a cofacial configuration and are engaged in a direct δ-type interaction, which is less effective than σ- and π-type interactions. At the same time, the 3d_*xy*_ orbital of vanadium has a σ-nonbonding character and can only delocalize on the thiocarboxylate ligands through weak π-type interactions with the O atoms, making the superexchange paths also inefficient, as previously reported for the Pt analogues.^18^ Overall, in both Pt and Pd derivatives, there will be a limited spin delocalization on the Group 10 metal via both direct and superexchange pathways (see below). As a consequence, the intradimer magnetic interaction is minimal in magnitude with respect to other PW complexes where the unpaired electrons are in σ-antibonding 3d orbitals with components along the Tr–M direction (e.g., 3d_*z* ^2^_).^8,16^ In the square thiobenzoate dimers (**2M**), the intradimer interaction is very weak but can still be clearly detected as a deviation from the straightforward magnetic response of two independent *S* = 1/2 spins. Fe^2+^, Co^2+^, and Ni^2+^ derivatives with a similar dimeric structure^8,9^ also exhibit a much-weakened coupling with respect to the corresponding staggered dimers. However, in that case, the magnetic analysis was complicated by single-ion zero-field splitting effects and orbital contributions to the susceptibility, and an intradimer *J* value could not be reliably determined.

Finally, while the small *J* values in **2M** may be biased by interdimer coupling (Figure S3),^18^ the results for **1M** clearly indicate a significantly stronger coupling in the Pd derivative, which is fully consistent with DFT predictions (see below and Table 2). It is noted that in the series of four eclipsed dimers [MTr­(SOCMe)_4_L] (Tr = Co, Ni; M = Pd, Pt; L = 3-nitropyridine) reported by Larsen et al.,^15^ replacement of Pt with Pd yields a remarkable decrease of 3d–3d coupling from 10–25 to 1–2 cm^–1^, a feature that so far lacks a sound theoretical explanation.

### 2.4 UV–Vis and NMR Investigations in Solution

Solutions of **1Pd** and **2Pd** were investigated by UV–vis spectroscopy and ^1^H NMR. The UV–vis absorption spectra in DCM solution are very similar for Pt and Pd compounds, with somewhat larger extinction coefficients in the Pd derivatives and the same weak double-peaked band in the 550–900 nm interval assigned to the vanadyl group absorption (Figure S6).

Like **1Pt**, **1Pd** is ^1^H NMR silent as a consequence of the strong paramagnetic effect of vanadium­(IV) on the nearby methyl protons.^18^ The ^1^H NMR spectra of **2Pd** in both CD_2_Cl_2_ (Figure S7) and THF-*d*_8_ (Figure S8) show a broad but clearly resolved peak at 8.70 ppm (Δν_1/2_ = 0.17 kHz), which is accompanied by an exceedingly broad signal at ca. 4 ppm (Δν_1/2_ > 2.5 kHz). Peaks at similar chemical shift values (8.50 and 4.4 ppm) are present in the ^1^H NMR spectrum of **2Pt** in CD_2_Cl_2_;^18^ their significantly smaller line width (89 Hz and 0.9 kHz) allowed integration and assignment to the *p*- and *m*-Ar protons, respectively. When THF-*d*_8_ was not dried with molecular sieves prior to use, the solution of **2Pd** gradually turned brownish on standing, and new, much narrower peaks emerged in the aromatic region of the ^1^H NMR spectrum. One set of signals can be tentatively assigned to the complex “[Pd­(SOCPh)_2_]”, while a second set is similar to the NMR pattern of thiobenzoic acid. At the same time, the water peak becomes broader and shifts from 1.52 to 2.65 ppm, indicating the solvation of vanadium­(IV) by water molecules (Figure S8). We contend that the presence of water triggers a structural rearrangement of the complex, which decomposes into separate V- and Pd-containing species. The latter then form insoluble brown precipitates of [Pd­(SOCPh)_2_]_*n*_ on the walls of the NMR tube.

The ^1^H DOSY NMR spectrum of **2Pd** in CD_2_Cl_2_ (Figure S9) allowed a direct estimation of the molecular weight (*MW*) in solution based on external calibration curves (ECCs).^46,47^ Following the same approach employed for **2Pt**,^18^ we obtained *MW* = 763 ± 236 and 872 ± 294 g mol^–1^, using the “dissipated spheres and ellipsoids” and merged calibration curves, respectively (details can be found in the Experimental Section).^47^ A third ECC reported by Byers et al.,^48^ which accounts for the presence of 3d transition elements, afforded *MW* = 812 ± 368 g mol^–1^. These results strongly suggest that **2Pd**, like **2Pt**, is monomeric in solution (expected *MW* = 722.08 g mol^–1^).

### 2.5 Electrochemistry

Cyclic voltammetry (CV) curves recorded at −15 °C on **1Pd** and **2Pd** and on their Pt analogues are shown in Figure 3.^18^ Both Pd PWs display one efficient quasi-reversible one-electron reduction/oxidation process, leading to a well-defined cathodic peak (A and D) with the corresponding anodic counterpart (B and E). The latter is, however, not very intense at low scan rates (*v* < 0.200 V s^–1^) and becomes evident and well-formed only by increasing *v* (*v* > 0.250 V s^–1^). This suggests that the reduced forms of **1Pd** and **2Pd** are somehow unstable and therefore only partially reoxidizable in the reverse scan. By contrast, the signals of the corresponding Pt complexes are stable and reversible at all scan rates. Table 3 presents the formal potentials (*E*°′ vs Fc^+^/Fc) in the series of four compounds, as well as the calculated thermodynamic contributions to *E*°′, extracted from the temperature-dependent measurements (see Supplementary Note 1).

**Figure 3:**
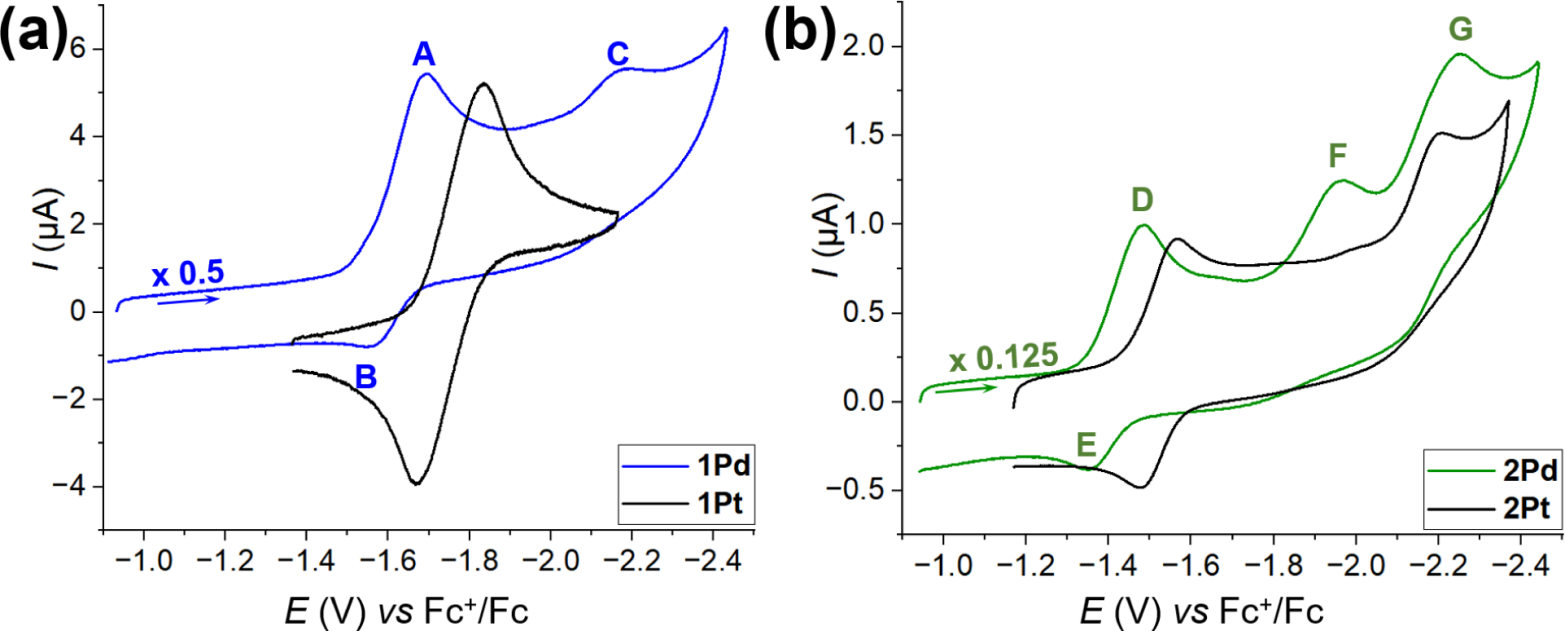
CV curves of **1M** (a) and **2M** (b). Glassy carbon (GC) working electrode, Ag wire quasi-reference electrode, −15 °C; for **1Pd** and **2Pd**: complex concentration 0.5 mM, 100 mM TBACl in DCM, scan rate 1 V s^–1^; for **1Pt** and **2Pt**: complex concentration 1.0 mM, 50 mM TBACl in DCM, scan rate 0.05 V s^–1^.^18^ The experimental conditions were adjusted for each sample to reach the best signal resolution.

**Table 3:** *E*°′ Values and Corresponding Thermodynamic Contributions in **1M** and **2M** ^a^

Compound	*E*°′(V)^d^	*E*_LUMO_ (eV)	Δ*S*°′_rc_ (J K^–1^ mol^–1^)	Δ*H*°′_rc_ (kJ mol^–1^)	*T*Δ*S*°′_rc_/*F* (V)^d^	–Δ*H*°′_rc_/*F* (V)
**1Pd** ^b^	–1.632	–1.964	–98 ± 4	132.2 ± 6.9	–0.262	–1.370
**1Pt** ^c^	–1.752	–1.813	–167 ± 5	125.9 ± 7.3	–0.446	–1.305
**2Pd** ^b^	–1.418	–2.123	–139 ± 4	101.0 ± 5.3	–0.372	–1.046
**2Pt** ^c^	–1.517	–2.116	–140 ± 3	110.3 ± 3.7	–0.374	–1.143

a Potential values and thermodynamic parameters are referred to the Fc^+^/Fc redox couple.

b 0.5 mM solution in DCM with 100 mM TBACl as base electrolyte, scan rate 1 V s^–1^.

c 1 mM solution in DCM with 50 mM TBACl as base electrolyte, scan rate 0.05 V s^–1^ (see ref 18).

d At −15 °C (*T* = 258 K); the error associated with the *E*°′ values is ±0.002 V.

The *E*°′ values span the range from −1.75 to −1.42 V and increase in the order **1Pt** < **1Pd** < **2Pt** < **2Pd**. In particular, each Pd-based PW is easier to reduce than the corresponding Pt derivative by ca. 100–120 mV. This trend correlates with the higher second ionization energy of Pd vs Pt (19.43 vs 18.56 eV).^49^ Furthermore, for the same Group 10 metal, the thiobenzoate derivative (**2M**) is easier to reduce than the thioacetate one (**1M**) by ca. 210–240 mV. This suggests that the R substituent on the thiocarboxylate ligand and the Group 10 metal act in a largely independent fashion in modulating the reduction potential of the PWs. According to the DFT calculations (see below), the LUMO is localized on the heavy metal in all the derivatives; therefore, the quasi-reversible peak can be attributed to the reduction M^2+^/M^+^. At more negative potentials, the thioacetate derivative **1Pd** displays a further cathodic peak (−2.190 V; C) which is not observed for **1Pt**, while **2Pd** shows two one-electron reduction peaks at −1.969 (F) and −2.249 V (G). These peaks have no evident anodic counterpart and are therefore due to irreversible reduction processes.

### 2.6 EPR Measurements

Continuous-wave (CW) and electron spin echo (ESE) detected EPR spectra of **1Pd** and **2Pd** were recorded at X-band frequency on 1 mM frozen solutions in CD_2_Cl_2_/toluene-*d*_8_ (1:1 v/v). The experimental EPR spectra (Figure 4) are typical of isolated vanadyl moieties and confirm that both complexes are present in solution as monomeric species. The spectra are dominated by the anisotropic hyperfine coupling of the electron spin (*S* = 1/2) with the nuclear spin of ^51^V (*I* = 7/2) that yields a characteristic eight-line pattern.

**Figure 4:**
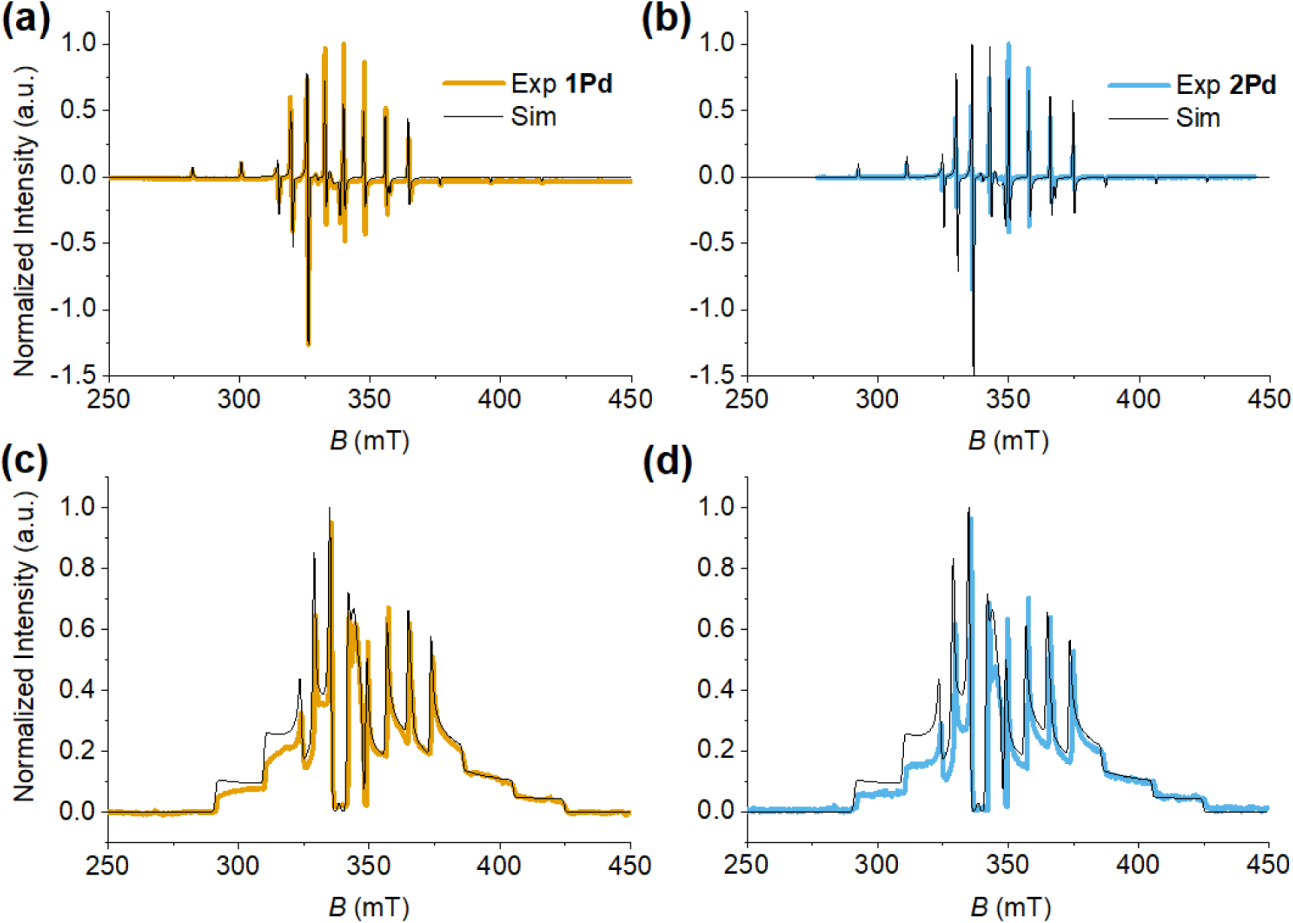
X-band CW- (a and b) and ESE-detected EPR spectra (c and d) of **1Pd** (a and c) and **2Pd** (b and d). CW spectra (a and b) were recorded at *T* = 77 K and ν = 9.4 GHz. ESE-detected spectra (c and d) were recorded at *T* = 10 K and ν = 9.7 GHz. The experimental/simulated spectra are drawn in yellow/black (a and c) or in blue/black (b and d) for **1Pd** and **2Pd**, respectively.

The spectra of **1Pd** and **2Pd** can be satisfactorily simulated (Figure 4a,b, black lines) assuming collinear **g̿** and ^V^ **A̿** matrices in the spin Hamiltonian (SH) of eq 1:

1 $$Ĥ=\mu_{B}B\cdot \mathrm{g̿}\cdot Ŝ+Ŝ\cdot^{V}\mathrm{A̿}\cdot {Î}_{V}$$

where *S* = 1/2 and *I*_V_ = 7/2. The SH parameters are in line with those reported for **1Pt** and **2Pt** ^18^ and are listed in Table 4. The same set of simulation parameters also successfully reproduces the ESE-detected spectra (Figure 4c,d, black lines). The SH parameters are consistent with the unpaired electron mainly localized in the 3d_*xy*_ orbital of vanadium­(IV). However, while the spectra of **1Pt** and **2Pt** show additional satellite lines arising from the hyperfine coupling with the naturally occurring fraction of ^195^Pt nuclei (*I* = 1/2, 34% abundance),^18^ no such hyperfine splitting is detected for ^105^Pd (*I* = 5/2, 22% abundance) due to the low nuclear *g* factor of Pd compared to that of Pt (*g*_Pd_= −0.257, *g*_Pt_= +1.219). A simulation analysis (see Supplementary Note 2) indicates a maximum coupling on the order of 5 MHz and suggests a lower spin density transfer to the Pd atom compared to the Pt analogue,^18^ consistent with DFT estimates (see below).

**Table 4:** Spin-Hamiltonian Parameters for **1Pd** and **2Pd** from X-Band CW-EPR Spectra and DFT Calculations^a^

	**1Pd**		**2Pd**	
Parameters	EPR^b^	DFT	EPR^b^	DFT
*g*_*x,y*_	1.982(1)	1.9751	1.982(1)	1.9744
*g*_*z*_	1.935(2)	1.9123	1.935(2)	1.9147
^V^ *A*_*x,y*_	196(2)	–272.802, –272.740	195(2), 200(2)	–267.458, –267.485
^V^ *A*_*z*_	517(3)	–609.372	517(3)	–602.460
^Pd^ *A*_*x*,*y*_	-	2.601	-	2.593
^Pd^ *A*_*z*_	-	5.338	-	5.084

a ^V^ *A*_*x*,*y*,*z*_ and ^Pd^ *A*_*x*,*y*,*z*_ describe the hyperfine and superhyperfine coupling with ^51^V and ^105^Pd nuclei, respectively, and are given in MHz; the experimental ^V^ *A*_*x*,*y*,*z*_ parameters are absolute values.

b Frozen CD_2_Cl_2_/toluene-*d*_8_ (1:1 v/v), 1 mM, 77 K.

The spin relaxation properties of **1Pd** and **2Pd** in solution were studied using the same standard pulse EPR protocols employed for the Pt analogues^18^ in order to ensure a meaningful comparison. All data were measured at a static magnetic field corresponding to the maximum intensity of the ESE-detected EPR spectrum (ca. 330 mT), which corresponds to the so-called powder position, where all possible molecular orientations are excited, providing the average contribution of all magnetic interactions (Zeeman and hyperfine).^50^ The temperature dependence of *T*_1_ and *T*_m_ was measured in the temperature interval 10–100 K, the upper temperature limit being dictated by the softening of the matrix. At all temperatures, the echo decay displays Electron Spin Echo Envelope Modulation (ESEEM) oscillations due to the deuterated solvent (see Figure S11).

Figure 5a reports the *T*_1_ and *T*_m_ values obtained by fitting the experimental traces with a biexponential function (see Figures S11 and S12, and Table S2). The *T*_m_ values were extracted by fitting the center of the echo decay traces. To test the robustness of the analysis, the time traces for both **1Pd** and its Pt analogue^18^ were also analyzed using mono- and stretched exponential functions (Table S3). At low temperatures (*T* < 50 K), the weighted average of the biexponential components agrees well with the monoexponential fit (e.g., 4.0 μs at 10 K vs a biexponential average of 3.5 μs for **1Pd**). Above 50 K, the decay is monoexponential. As a matter of fact, the biexponential fit converges to identical values for the slow and fast components.

**Figure 5:**
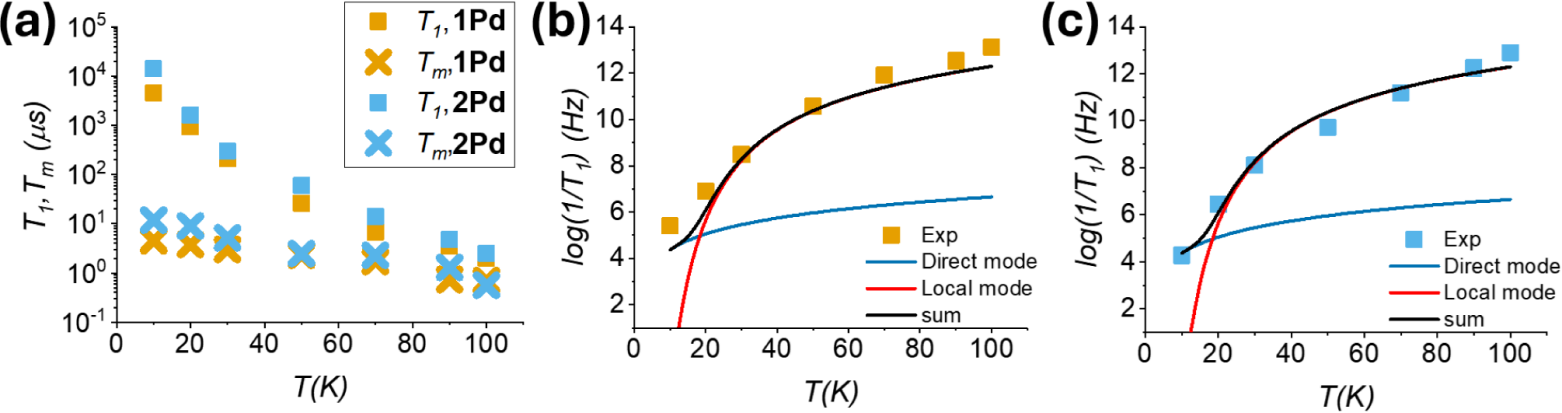
(a) Temperature dependence of *T*_1_ (squares) and *T*_m_ (crosses) for **1Pd** (yellow) and **2Pd** (blue). Fit of the dependence of *T*_1_ vs temperature for **1Pd** (b) and **2Pd** (c) using eq 2.

Furthermore, given the pronounced ESEEM modulations in the low-temperature decay traces, the fits were also performed considering the maxima of the modulation (Figures S13 and S14). This does not alter the overall trend, but leads to a systematic increase in *T*_m_ by ∼10% when comparing the weighted average value obtained from the biexponential fit with the corresponding values obtained from mono- and stretched-exponential fits. Taking into account other sources of uncertainty,^50^ we estimate the overall uncertainty in both *T*_1_ and *T*_m_ of the order of 20%. In the temperature interval 10–90 K, the slow component of a biexponential fit affords *T*_m_ values of 4.5–0.7 μs in **1Pd** and 12–1.3 μs in **2Pd**. As previously observed for the **1Pt** and **2Pt** analogues,^18^ **2Pd** generally displays slightly longer relaxation times than **1Pd**. It has been shown in multiple systems^50,51^ that the rotation of the methyl groups has a detrimental effect on *T*_m_. The bigger and sterically hindered phenyl substituents, on the other hand, have a smaller effect. The effect of the Group 10 metal is negligible as shown by the value of *T*_m_ measured at 10 K, which goes from 6.1 to 4.5 μs and from 11 to 12 μs upon replacing Pt with Pd in **1M** and **2M**, respectively.

Figure 5a also shows a comparison of the dependence of *T*_1_ on temperature for **1Pd** and **2Pd**. The experimental points correspond to the slow component of a biexponential fit, which is usually taken as a measure of the actual *T*_1_.^26^ In line with *T*_m_, **2Pd** displays longer *T*_1_ in the whole temperature range. For instance, at 10 K, the maximum values are of ca. 4.5 and 14 ms in **1Pd** and **2Pd**, respectively. The temperature dependence of the relaxation times proves that the values for Pd are very similar to those of Pt (4.0 and 11 ms for **1Pt** and **2Pt**, respectively, at 10 K), indicating that the replacement of Pt with Pd does not substantially affect the spin–lattice relaxation, even though the SOC constant of Pd is approximately three times smaller than that of Pt.^52,53^ A plausible explanation is related to the minute spin density delocalization (ca. 1%) on the Group 10 metal.

To assess the effect of the lighter Group 10 metal on molecular vibrations and, in turn, on the spin–lattice relaxation, the temperature dependence of 1/*T*_1_ was analyzed using the same model employed for the Pt counterparts.^18^ This is important to ensure a meaningful comparison between the Pd and Pt complexes. The model (eq 2) assumes that low-energy optical vibrations are responsible for Raman-like processes and also includes a direct mechanism contribution active at low temperature:^54,55^

$$\frac{1}{T_{1}}=A_{\mathrm{dir}}\frac{e^{\hbar \omega_{\mathrm{mw}}/k_{B}T}}{e^{\hbar \omega_{\mathrm{mw}}/k_{B}T}-1}+A_{\mathrm{loc}}\frac{e^{\hbar \omega_{\mathrm{loc}}/k_{B}T}}{{\left(e^{\hbar \omega_{\mathrm{loc}}/k_{B}T}-1\right)}^{2}}$$ 2

where ω_mw_ is the working microwave frequency (ω_mw_/2π = 9.74 GHz), and ω_loc_ is the frequency of an average effective vibration responsible for the so-called Raman process. The experimental data were fitted, leaving as free parameters only ω_loc_ and the coefficients *A*_dir_ and *A*_loc_, which quantify the effectiveness of each relaxation process. The fitted curves are shown in Figure 5b,c, and the parameters are reported in Table S4. For the two complexes, the model yields an average effective vibration with a frequency of 110–120 cm^–1^. This result agrees with DFT simulations. The computed low-energy (<200 cm^–1^) vibrational frequencies for **1Pd** and **2Pd** show a strong similarity with those calculated for the Pt analogues (see Tables S6, S7, and ref 18); even in this case, the thiobenzoate derivative presents a larger number of vibrations below 50 cm^–1^, which are localized on the phenyl rings. Furthermore, in the examined frequency range, the vibrational modes of the thioacetate complex **1Pd** have slightly lower energy than those of **1Pt**, and the same trend is visible when comparing the vibrational frequencies above 80 cm^–1^ for the two thiobenzoate derivatives.

The observation of an electron spin echo, per se, demonstrates the presence of electron spin coherence. However, in literature, this is further substantiated through Rabi nutation experiments, which were performed at 70 K, demonstrating the possibility of placing the spins in any arbitrary superposition of states (Figure S15).

### 2.7 DFT Calculations

DFT calculations were first performed to evaluate the sign and magnitude of intradimer magnetic interactions in the solid state (Table 2). Within the Broken Symmetry (BS) approach, the simulations for **1Pd** showed an antiferromagnetic intradimer coupling with *J =* 7.44 cm^–1^, hence slightly larger than calculated for **1Pt** at the same level of theory (6.55 cm^–1^).^18^ A much smaller antiferromagnetic interaction is expected in **2Pd** (0.79 cm^–1^) and **2Pt** (0.47 cm^–1^),^18^ again with a small increase of antiferromagnetic coupling when replacing Pt with Pd. Although DFT calculations slightly overestimate most *J* values, the experimental data are largely consistent with these theoretical predictions (Table 2). Since intradimer geometrical parameters (M···M′ distances, twisting angles, etc.) are almost the same in the Pd and Pt derivatives (Table 1), the reason behind the observed trend lies in the electronic structure of Pd. The 4d orbitals are less diffuse than the 5d orbitals and lead to a larger overlap and a larger antiferromagnetic contribution. Worth noting is the opposite trend that would be suggested by the extent of spin delocalization on the Group 10 metal. According to Löwdin spin population analysis, the spin density on Pd is 0.012 e^–^ in **1Pd** and 0.010 e^–^ in **2Pd**. These values are lower than those computed for the platinum derivatives (0.014 and 0.011 e^–^ on Pt in **1Pt** and **2Pt**, respectively^18^), in agreement with the EPR findings and the longer V–Pd distances, which lead to a less effective spin delocalization through V–Pd δ interaction. Even if full validation of such small variations would require a proper benchmark on a series of different compounds, the overall trend nicely correlates with the observed exchange constants.

DFT calculations were also used to relate the reduction potentials for the two compounds to the energies of their LUMO orbitals, which are −1.964 eV (**1Pd**) and −2.123 eV (**2Pd**). The reversed sign energy of the LUMO represents the electron affinity within Koopmans’ theorem. **2Pd** should then be easier to reduce than **1Pd**, as found for the two Pt analogues^18^ and confirmed experimentally. In addition, the greater propensity of **1Pd** and **2Pd** to undergo reduction with respect to their Pt counterparts agrees with their computed larger electron affinity (Table 3). It is also worth stressing that the LUMO of **1Pd** and **2Pd**, as that of their Pt counterparts,^18^ is localized on the Group 10 metal and shows a large 4d_*x* ^2^–*y* ^2^_ component (37.4 and 16.9% in **1Pd** and **2Pd**, respectively, according to Löwdin population analysis).

Finally, to account for the absence of a detectable hyperfine coupling with ^105^Pd, we computed the ^105^Pd hyperfine tensor (^Pd^ **A̿**) in each Pd derivative (Table 4) and its composition in terms of Fermi contact, dipolar, and SOC contributions (Table S5). The overall hyperfine coupling amounts to ca. 5 MHz and is thus much smaller than in the Pt analogues.^18^ This estimate explains the absence of a detectable hyperfine coupling with ^105^Pd and is consistent with the simulation analysis presented in the Supplementary Note 2. As a direct consequence of the lower SOC in Pd, the computed ^Pd^ **A̿** tensor is also much less anisotropic than ^Pt^ **A̿**. For the same reason, the fraction of isotropic coupling arising from SOC is very different: 4.9–5.2% in **1Pd** and **2Pd**, and 15.3–16.7% in **1Pt** and **2Pt** (the remaining fraction originates from Fermi contact interaction).

## 3 Conclusions

Thiocarboxylate PW structures [PdVO­(SOCR)_4_] with R = Me (**1Pd**) and Ph (**2Pd**) contain an O-bonded vanadyl ion and an S-bonded Pd^2+^ ion, and have proven synthetically accessible despite the dual coordination nature of Pd^2+^, which can interact with both soft and hard donor atoms. Molecular and crystal structures are closely similar to those of the corresponding Pt derivatives **1Pt** and **2Pt**,^17^ with formation of staggered dimers supported by short Pd···Pd′ contacts (**1Pd**) or square dimers featuring two reciprocating Pd···S′ interactions (**2Pd**). The Group 10 metal has a small but significant effect on the strength of magnetic coupling within the staggered dimers via the metallophilic contact. The interaction is invariably antiferromagnetic, with *J* = 4.7 and 5.2 cm^–1^ in **1Pt** and **1Pd**, respectively (*J* **S**_1_·**S**_2_ convention). The slightly larger coupling promoted by the Pd···Pd′ bridge is correctly reproduced by DFT calculations within the BS approach.

The dimers dissociate into monomers in organic solution, as evidenced by ^1^H DOSY NMR and CW-EPR spectra. CV demonstrated that all four complexes undergo a quasi-reversible one-electron reduction, which is mostly centered on the Group 10 metal according to DFT studies. This reduction is experimentally found (and theoretically predicted to be) easier in Pd than in Pt derivatives, and in thiobenzoates than in thioacetates.

The X-band EPR spectra recorded in a frozen CD_2_Cl_2_/toluene-*d*_8_ matrix display a well-resolved hyperfine pattern due to the interaction of the *S* = 1/2 spin with the ^51^V nucleus (*I* = 7/2, ∼100% abundance). The minority fraction of magnetically active ^105^Pd isotope (*I* = 5/2, 22% abundance) gives no detectable hyperfine splitting of the X-band EPR lines, unlike the ^195^Pt isotope in the Pt derivatives (*I* = 1/2, 34% abundance).^18^ Simulation analysis shows that this is due to the combined effect of a decreased spin delocalization on Pd (confirmed by DFT) and the approximately 5-fold smaller nuclear *g*-factor of ^105^Pd vs ^195^Pt. Coherence times measured by X-band pulsed EPR spectroscopy are comparable to those found in the Pt analogues in the same conditions, with thiobenzoate derivatives displaying systematically longer values (11–12 vs 4–6 μs at 10 K).^18^ Apparently, the largely decreased SOC constant of the outermost d electrons of Pd has no effect on quantum coherence, presumably because of the minute (ca. 1%) spin density delocalized on the proximal Group 10 metal.

Our results demonstrate that thiocarboxylate-based PWs containing vanadyl ion and a diamagnetic Group 10 metal can be chemically tailored by modifying both the R substituent on the thiocarboxylate ligand and the ancillary heavier metal. This enables a fine-tuning of properties like molecular size, hydrophobicity, solubility, redox potentials, and intradimer magnetic coupling, while preserving quantum coherence. Further possibilities can be envisaged, like exploiting the different radial extension of 4d vs 5d orbitals to modulate surface-molecule interaction energy and electronic coupling.

## 4 Experimental Section

### 4.1 General Methods

Anhydrous DCM, *n*-hexane, acetone, D_2_O (99.9 atom % D), CD_2_Cl_2_ (99.8 atom % D), THF-*d*_8_ (99.5 atom % D), and toluene-*d*_8_ (99.5 atom % D) were used as received. MeOH was predried with activated 3 Å molecular sieves (MS), then degassed by purging with dry nitrogen flux, distilled from Mg­(OMe)_2_, and stored over activated 3 Å MS in a nitrogen-filled Schlenk vessel.^56^ THF was dried over Na, then distilled over Na with the addition of benzophenone under a dry nitrogen atmosphere, and stored over activated 4 Å MS prior to use. **1Pd** was synthesized under nitrogen using standard Schlenk techniques. Compound **2Pd** is air-sensitive in solution and was recrystallized and stored inside a nitrogen-filled MBraun glovebox. Both deuterated and regular solvents for use in the glovebox were degassed by three *freeze–pump–thaw* cycles and stored over activated 4 Å MS. The water content in commercially available VOSO_4_·*n*H_2_O was determined by elemental analysis (EA) as *n* = 3.6. KSOCMe (98%) and K_2_PdCl_4_ were used in commercially available form. NaSOCPh was prepared by reaction of HSOCPh (93%, contaminated with benzoyl disulfide according to ^1^H NMR) with NaHCO_3_ in water, and its purity was checked by ^1^H NMR in D_2_O, EA, and IR spectroscopy.

EA (CHN) was performed using a Thermo Fisher Scientific Flash 2000 analyzer. IR spectra were measured on a Jasco 4700 FT-IR spectrometer in ATR mode with a resolution of 1 cm^–1^. ^1^H NMR spectra of **1Pd** and **2Pd** were collected in CD_2_Cl_2_ or THF-*d*_8_ (*C* = 0.01 M) at 298 K on an Avance 400 spectrometer from Bruker Biospin. Chemical shifts (δ, ppm) are expressed downfield from TMS and referenced to the residual proton resonances of the solvent (CD_2_Cl_2_: δ = 5.32; THF-*d*_8_: δ = 1.72 [CHD­(3,4)])^57^ or to TMS as an internal standard. ^1^H DOSY NMR measurements on **2Pd** were carried out with a *ledbpgp2s* sequence (Bruker library) using bipolar gradient pulses.^58^ The diffusion time (or *big delta*) and the gradient length (or *small delta*) were tuned to 0.03 s and 800 μs, respectively, using the monodimensional sequence *ledbpgp2s1d* (Bruker library). The signal decay was fitted with a single exponential function using Bruker Dynamic Center software (version 2.8.3). Diffusion coefficients were normalized using TMS as an internal reference following the procedure described by Stalke et al.^47^ Alternatively, the residual proton signal of CD_2_Cl_2_ was used as a reference for the ECC developed by Byers et al.^48^ The absolute errors on estimated *MW*s were calculated as reported by Stalke et al., who showed that these errors are largely determined by the uncertainties on ECC parameters.^59^ Electronic absorption spectra were collected on ca. 3 × 10^–3^ M solutions in DCM using a Jasco V-770 UV–vis–NIR spectrometer operating in double-beam mode (optical path length *l* = 0.1 cm).

### 4.2 Synthesis of [PdVO­(SOCMe)_4_] (**1Pd**)

Solid VOSO_4_·3.6H_2_O (14.8 mg, 0.0649 mmol), K_2_PdCl_4_ (21.4 mg, 0.0656 mmol), and KSOCMe (29.8 mg, 0.261 mmol) were introduced into a Schlenk vessel, which was then evacuated and refilled with nitrogen. MeOH (5 mL) was added via a cannula under a nitrogen atmosphere, and the mixture was stirred for 18 h. Upon the course of the reaction, it turned into a brown suspension. The solvent was evaporated on a vacuum line, then the Schlenk vessel was opened to the air, and the emerald-green soluble product was extracted with 4 mL of DCM, while other products were filtered off. Evaporation gave a light green powder (20.7 mg, 67% yield based on Pd). EA (C_8_H_12_O_5_PdS_4_V, 473.80) calcd. C 20.28, H 2.55%; found C 21.08, H 2.69%. To obtain X-ray quality crystals, the compound was redissolved in DCM (0.5 mL), and *n*-hexane (6 mL) was layered above the solution. Upon standing, large emerald-green crystals grew up. EA (crystals, C_8_H_12_O_5_PdS_4_V, 473.80) calcd. C 20.28, H 2.55%; found C 20.28, H 2.37%. IR (Figure S5, ν̃ _max_, cm^–1^): 1540 s, 1516 m sh, 1502 m, 1473 m, 1414 m br, 1346 m, 1238 w, 1144 s, 1100 w sh, 998 s, 930 w sh, 723 s, 672 w sh, 539 w, 509 s, 447 s. UV–vis (Figure S6, DCM, 3.5 × 10^–3^ M): λ_max_, nm (ε, M^–1^cm^–1^) ∼608 (26), 714 (26).

### 4.3 Synthesis of [PdVO­(SOCPh)_4_] (**2Pd**)

NaSOCPh (102.2 mg, 0.6380 mmol) and K_2_PdCl_4_ (51.8 mg, 0.159 mmol) were separately dissolved in 3 and 5 mL of water, respectively. Upon vigorous stirring, the solution of K_2_PdCl_4_ was dropwise added to the solution of NaSOCPh over 10 min in such a manner that no precipitate formed. The solution turned orangish-red. VOSO_4_·3.6H_2_O (36.4 mg, 0.160 mmol) was dissolved in water (2 mL) and added immediately after the addition of K_2_PdCl_4_ was over, resulting in the formation of an ocher precipitate. The reaction was allowed to proceed for 1 h, and the precipitate was collected on a glass filter and extensively washed with water. The precipitate on the filter was then washed with small portions of DCM that caused the dissolution of a red component, leaving only a grassy-green solid on the filter (acetone can be used as an alternative to DCM). The filter was dried in a vacuum until a constant mass. Crude yield 63.0 mg (56.5% based on Pd). The green precipitate was dissolved in hot THF, the solution was passed through the same filter, and evaporated to dryness. The subsequent recrystallization steps were carried out inside the glovebox. THF (0.5 mL) was added to the precipitate, and the suspension was allowed to stand for a few days. The compound recrystallized in the form of grassy-green plates. A slightly brownish mother liquid was decanted, and a fresh portion of THF was added. The crystals were identified by X-ray diffraction as THF solvate [PdVO­(SOCPh)_4_]·THF (**2Pd**·THF). After a few days, a green mother liquid was decanted, the crystals were briefly washed with 1 mL of *n*-hexane and dried in a flow of nitrogen, giving 41.3 mg (36%) of the pure product. EA better agrees with formula [PdVO­(SOCPh)_4_]·0.2THF (C_28.8_H_21.6_O_5.2_PdS_4_V, 736.50): calcd. C 46.97, H 2.95%; found C 46.76, H 3.23%. A portion of the product was recrystallized from DCM in the same fashion, by leaving the powder under a small amount of solvent. The crystals obtained from this batch were identified by X-ray diffraction as DCM solvate [PdVO­(SOCPh)_4_]·DCM (**2Pd**·DCM). The mother liquid was decanted, and the crystals were washed with *n*-hexane and dried in a flow of nitrogen. EA results agree with a solvent-free compound (C_28_H_20_O_5_PdS_4_V, 722.08): calcd. C 46.57, H 2.79%; found 46.68, H 2.82%. IR (Figure S5, ν̃ _max_, cm^–1^): 1591 w, 1504 m, 1462 m, 1435 m, 1338 w, 1307 w, 1214 s, 1172 s, 1096 w, 1072 w, 1006 s, 955 s, 928 m sh, 894 w sh, 841 w, 770 s, 724 s, 682 s, 642 s, 614 w, 578 s, 512 s, 503 m, 484 w, 438 s. UV–vis (Figure S6, DCM, 3.1·10^–3^ M): λ_max_, nm (ε, M^–1^cm^–1^) ∼340 (8.8 × 10^4^), 620 (51), 711 (50).

^1^H NMR (Figure S7, 400.13 MHz, CD_2_Cl_2_) δ 8.70 (br s, 4H; *p*-C_6_ *H*_5_), ∼4 (vbr s, 8H; *m*-C_6_ *H*_5_).

^1^H NMR (Figure S8, 400.13 MHz, THF-*d*_8_) δ 8.69 (br s, 4H; *p*-C_6_ *H*_5_), ∼4 (vbr s, 8H; *m*-C_6_ *H*_5_).

### 4.4 X-ray Crystallography

Single-crystal X-ray diffraction data on **1Pd**, **2Pd**·THF, and **2Pd**·DCM were collected on a Bruker-Nonius X8APEX diffractometer, equipped with a Mo Kα generator, an area detector, and a Kryoflex cryostat for data collection at 200 K. One of the components of an epoxy glue was used for crystal handling, selection, and mounting without significant loss of any lattice solvent. Matrix frames and data collection were done with APEX2 v1.0-22 software, while data reduction was performed with SAINT v7.06A program and was followed by scaling and multiscan absorption correction using SADABS v2.10.^60^ The structures were solved by direct methods (SIR92)^61^ and refined by full matrix least-squares methods on *F*_o_^2^ using SHELXL-2018/3 ^62^ program and WINGX^63^ v2020.2 suite. Unless otherwise noted, all non-hydrogen atoms were assigned anisotropic displacement parameters while H atoms were treated isotropically, with *U* = 1.5*U*_eq_(C) for methyl groups and *U* = 1.2*U*_eq_(C) for the other H atoms. In **1Pd**, CH_3_ groups were subject to rotating group refinement (AFIX 37). In **2Pd**·THF and **2Pd**·DCM, all aromatic H atoms were fully refined, while methylene hydrogens were placed in geometrically idealized positions and allowed to ride on the parent C atom. Atom C31 of the lattice THF molecule in **2Pd**·THF required split-atom refinement with a common isotropic displacement parameter for the two components (ca. 60:40).

Deposition Numbers 2443816–2443818 contain the supplementary crystallographic data for this paper. These data are provided free of charge by the joint Cambridge Crystallographic Data Centre and Fachinformationszentrum Karlsruhe Access Structures service.

### 4.5 Magnetic Measurements

The magnetic measurements were carried out with a Quantum Design MPMS-XL SQUID magnetometer and a PPMS-9 susceptometer housed at the Center de Recherche Paul Pascal (Pessac, France). The MPMS-XL instrument works between 1.85 and 400 K with applied static fields (*H*) ranging from −7 to 7 T. In order to avoid loss of lattice solvent, crystals of **2Pd**·THF stored under the mother liquor were collected by filtration and sealed in a polypropylene bag (typical size ≈ 2 × 1 × 0.02 cm) under an argon atmosphere just before measurement. The magnetic susceptibility was evaluated as χ = *M*/*H*, where *M* is the magnetization. Data reduction for **1Pd** and **2Pd**·THF (12.76 and 25.40 mg, respectively) was carried out assuming *MW* = 473.8 and 794.2 g mol^–1^, corresponding to the formulas [PdVO­(SOCMe)_4_] and [PdVO­(SOCPh)_4_]·THF, respectively, and a diamagnetic correction estimated as −0.5 *MW*·10^–6^ cm^3^ mol^–1^. Note that the use of an *MW* corresponding to the unsolvated **2Pd** yields unacceptably low *g* values in the fit. The ac susceptibility measurements down to 1.85 K were performed with the MPMS-XL and PPMS-9 systems using an oscillating field from 1 to 6 Oe for frequencies between 1 Hz and 10 kHz in a zero-dc field and up to 1 T. In the available temperature and frequency ranges, the samples do not display any observable slow relaxation of the magnetization.

### 4.6 Electrochemistry

A potentiostat/galvanostat PARSTAT mod. 2273A (EG&G PAR, Oak Ridge, USA) was used for CV measurements set up in a nitrogen-filled glovebox equipped with a cooling bath of dry *n*-heptane and a coldfinger cryostat for low-temperature operations. Experiments were carried out in the temperature range from −30 to −5 °C in steps of 5 °C and at different scan rates (*v* = 0.02–5 V s^–1^) using a cell for small-volume samples (2 mL). A 1 mm diameter GC disk (PAR), a Pt wire, and an Ag wire were used as working, counter, and quasi-reference electrodes, respectively. The GC electrode was cleaned following a previously reported procedure.^64,65^ The measurements were carried out in DCM on 0.5 mM (1 mM) solutions of **1Pd** and **2Pd** (**1Pt** and **2Pt**) using 100 mM (50 mM) TBACl as a supporting electrolyte. A careful feedback correction minimized the ohmic drop between the working and the reference electrodes. The ferrocenium/ferrocene (Fc^+^/Fc) redox couple (in DCM, *E*°′ = 0.460 V vs SCE and 0.946 ± 0.005 V vs the Ag wire quasi-reference electrode used in our experiments) was used to calibrate the potential of the quasi-reference electrode.^66^ For the reversible CV signals, the formal potential value (*E*°′) of the electron transfer process was calculated as the semisum of the cathodic and anodic peak potentials, *E*°′ = *E*_1/2_ = (*E*_pc_ + *E*_pa_)/2. The peak currents of all the signals were found to be proportional to the square root of *v* (not shown), indicating diffusion-controlled electron transfer processes. All the reported *E*°′ values are referred to the Fc^+^/Fc redox couple. Experiments with the addition of ferrocene standard were repeated at each temperature. Variable temperature experiments were carried out in an isothermal cell configuration in which the temperature of the reference and working electrodes was always the same. For this experimental configuration, the reduction entropy referenced to the Fc^+^/Fc redox couple (Δ*S*°′_rc_) is given by eq 3:

$$\Delta {S^{{}^{\circ}\prime }}_{\mathrm{rc}}={S^{{}^{\circ}\prime }}_{\mathrm{red}}-{S^{{}^{\circ}\prime }}_{\mathrm{ox}}=nF{\left(\partial E^{{}^{\circ}\prime }/\partial T\right)}_{P,ni}$$ 3

Thus, Δ*S*°′_rc_ can be calculated from the slope of the *E*°′ vs *T* plot, when it is linear. Under the same conditions, the enthalpy change (also referenced to the Fc^+^/Fc redox couple, Δ*H*°′_rc_) was obtained from the Gibbs–Helmholtz equation, namely from the *E*°′/*T* vs 1/*T* plot. Each experiment was repeated at least five times. The CV curves remained stable over time in the investigated temperature range, and *E*°′ values were found to be reproducible within ± 0.002 V.

### 4.7 EPR Measurements

All EPR experiments were performed on frozen solutions using a CD_2_Cl_2_/toluene-*d*_8_ (1:1 v/v) mixture as a solvent. The samples were prepared in an argon-filled glovebox with oxygen and water levels below 0.5 ppm. The final concentration of the **1Pd** and **2Pd** samples was 1 mM. The EPR tubes were sealed with PTFE tape before removal from the glovebox.

CW-EPR spectra were recorded at X-band frequency (ν ≈ 9.40 GHz) on a Bruker EMX spectrometer equipped with an SHQ cavity. Low-temperature measurements were obtained using a finger Dewar working at 77 K. The experimental parameters were: microwave power 1 mW, modulation amplitude 0.4 mT, conversion time 20 ms.

Pulsed EPR spectra were also recorded at X-band frequency (ν ≈ 9.74 GHz) on a Bruker Elexsys E580 spectrometer equipped with a dielectric ring resonator (ER4118X-MD5) housed in a cryogen-free variable temperature cryostat (Cryogenic Ltd.). During the measurements, the resonator was overcoupled to minimize ringdown effects due to the application of the intense microwave pulses. The ESE-detected EPR spectra were measured at *T* = 10 K, integrating the whole spin echo generated by the Hahn echo sequence (π/2−τ–π–τ–echo, with τ = 200 ns and π/2 = 16 ns) as a function of the applied magnetic field. Spin coherence time traces (*T*_m_) were measured by integrating the top of the spin echo (40 ns) generated by the Hahn echo sequence as a function of τ. Attention was paid to set the shot repetition time (SRT) at least five times larger than *T*_1_ in order to avoid artifacts related to partial saturation of the echo signal. Spin–lattice relaxation time (*T*_1_) traces were recorded by integrating the whole spin echo generated by the inversion recovery sequence (π–*t*_w_–π/2−τ–π–τ–echo, with τ = 200 ns and π/2 = 16 ns) as a function of the waiting time *t*_w_. Rabi oscillations were measured by integrating the whole spin echo generated by the sequence *t*_nut_–*t*_w_–π/2−τ–π–τ–echo as a function of the length of the nutation pulse *t*_nut_ (from 0 to 2046 ns in steps of 2 ns), while keeping *t*_w_ = 4 μs and τ = 200 ns fixed. The linear dependence with respect to the applied microwave field was demonstrated by recording Rabi traces as a function of the microwave power attenuation at 1, 7, and 14 dB for **1Pd** and 0, 6, and 12 dB for **2Pd**. The primary *T*_1_ and *T*_m_ time traces were fitted according to a biexponential model, described by eqs 4 and 5.

4 $$I\left(t_{w}\right)=y_{0}+A_{\mathrm{fast}}\;e^{-\frac{t_{w}}{T_{1,\mathrm{fast}}}}+A_{\mathrm{slow}}\;e^{-\frac{t_{w}}{T_{1,\mathrm{slow}}}}$$

5 $$I\left(2\tau \right)=y_{0}+A_{\mathrm{fast}}\;e^{-\frac{2\tau }{T_{m,\mathrm{fast}}}}+A_{\mathrm{slow}}\;e^{-\frac{2\tau }{T_{m,\mathrm{slow}}}}$$

The *T*_m_ values were extracted by fitting the center of the echo decay traces. To test the robustness of the analysis, the time traces for both **1Pd** and its Pt analogue were analyzed using also mono- (eq 6) and stretched exponential (eq 7) functions.

6 $$I\left(2\tau \right)=y_{0}+A\;e^{-\frac{2\tau }{T_{m}}}$$

7 $$I\left(2\tau \right)=y_{0}+A\;e^{-{\left(\frac{2\tau }{T_{m}}\right)}^{\beta }}$$

All simulations were performed using the EasySpin 6.0.6 package working in Matlab.^67^

### 4.8 DFT Calculations

All the calculations were performed with the ORCA 6.0.1 quantum chemistry package.^68^ The X-ray structures of **1Pd** and **2Pd** were optimized in the gas phase by DFT. Solvent (toluene) effects were included within the CPCM model.^69^ We used the PBE0 hybrid exchange-correlation functional^70^ along with D3 atom pairwise dispersion corrections with BJ damping.^71^ Def2-TZVPP basis set^72^ was employed for all the atoms. For palladium, we used an effective core potential^73^ to describe the inner core electrons. For the optimization runs, “TightOpt” convergence criteria were set. Afterward, we ran analytical frequency calculations on the final optimized structures to verify that the geometry is a true local minimum of the potential energy surface.

The calculation of single-ion SH parameters, i.e., *g*-tensor and hyperfine coupling, was performed at the same level of theory on the final optimized structure with Douglas-Kroll-Hess (DKH) relativistic corrections and basis sets (DKH-def2-TZVPP). SARC basis sets were employed for palladium.^74^

The intradimer exchange interaction was computed in dimer models. The models were built using two molecular neighboring units extracted from the crystal structure, as in our previous work on the Pt derivatives.^18^ The B3LYP functional^75^ was chosen with def2-TZVPP basis for all atoms.

The isotropic magnetic-coupling constants were extracted within the BS approach,^76^ as developed by Noodleman. Such a method enables the calculation of magnetic interactions using DFT without the need to employ computationally expensive multiconfigurational approaches. Therefore, single-point calculations on both the triplet and the BS state were performed. Afterward, we mapped the spin states on the Heisenberg–Dirac–Van Vleck Hamiltonian *Ĥ*_HDVV_ *= J* **Ŝ**_1_·**Ŝ**_2_, where *S*_1_ = *S*_2_ = 1/2, and computed the value of the superexchange constant *J* with the following formula:

8 $$J=\left(E_{\mathrm{HS}}-E_{\mathrm{BS}}\right)/\left(2S_{1}S_{2}\right)$$

In eq 8, *E*_HS_ is the energy of the high-spin (HS) triplet state (total spin *S* = 1) and *E*_BS_ is the energy of the BS state (nontotal symmetric *S* = 0 singlet).

## Supplementary Material





## Notes

Additional crystallographic information and structural diagrams, IR, UV–vis, and NMR spectra, additional electrochemical data, details on EPR spectroscopy and magnetic measurements (PDF)
Computational data including Cartesian coordinates of DFT optimized structures (XYZ)
