# Supplementary material for: Oxovanadium(IV) Thiocarboxylate Paddlewheels Containing Ancillary Group 10 Metals: A Comparative Study on Pd and Pt Derivatives
Source: Inorg Chem. 2025 Dec 19;65(1):638–51. doi: 10.1021/acs.inorgchem.5c04835 (PMC12801318; doi:10.1021/acs.inorgchem.5c04835)
Supplement: Supplementary file 1 [file ic5c04835_si_001.pdf]

Supporting Information

Oxovanadium(IV) thiocarboxylate paddlewheels containing ancillary Group 10 metals: a comparative study on Pd and Pt derivatives

Olga Mironova,[a]* Giacomo Bellini,[a] Alessio Nicolini,[a] Manuel Imperato,[a,b] Antonio Ranieri,[c] Marco Borsari,[a] Matteo Briganti,[d] Rodolphe Clérac,[e] Mathieu Rouzières,[e] Enrico Salvadori,[f] Maria Chiara Pagliero,[f] Mario Chiesa,[f] and Andrea Cornia[a]*

Table of contents

1. X-Ray diffraction S2
2. IR, UV-Vis, and NMR spectroscopy S5
3. Cyclic voltammetry S8
    Supplementary Note 1 .... S8
4. EPR spectroscopy S9
    Supplementary Note 2 .... S12
5. DFT calculations S14
6. References S16

---

[a]Dipartimento di Scienze Chimiche e Geologiche e UdR INSTM, Università degli Studi di Modena e Reggio Emilia, via G. Campi 103, 41125 Modena, Italy. *E-mail: acornia@unimore.it, mironovaoa.nsk@gmail.com

[b]Dipartimento di Scienze Fisiche, Informatiche e Matematiche, Università degli Studi di Modena e Reggio Emilia, via G. Campi 213/A, 41125 Modena, Italy

[c]Dipartimento di Scienze della Vita, Università degli Studi di Modena e Reggio Emilia, via G. Campi 103, 41125 Modena, Italy

[d]Dipartimento di Chimica "Ugo Schiff" e UdR INSTM, Università degli Studi di Firenze, via della Lastruccia 3, 50019 Sesto Fiorentino, FI, Italy

[e]Univ. Bordeaux, CNRS, Centre de Recherche Paul Pascal, CRPP, UMR 5031; 33600 Pessac, France

[f]Dipartimento di Chimica e NIS Centre, Università degli Studi di Torino, via P. Giuria 7, 10125 Torino, Italy

https://pubs.acs.org/doi/10.1021/acs.inorgchem.5c04835

1. X-Ray diffraction

Table S1. Crystal data and refinement parameters for **1Pd**, **2Pd**·DCM, and **2Pd**·THF.

	**1Pd**	**2Pd**·DCM	**2Pd**·THF
Radiation	MoKα (λ = 0.71073 Å)	MoKα (λ = 0.71073 Å)	MoKα (λ = 0.71073 Å)
Chemical formula	$C_8H_{12}O_5PdS_4V$	$C_{29}H_{22}Cl_2O_5PdS_4V$	$C_{32}H_{28}O_6PdS_4V$
Formula weight	473.76	806.94	794.12
T (K)	200(2)	200(2)	200(2)
Crystal size (mm^3)	0.35 × 0.07 × 0.02	0.41 × 0.23 × 0.18	0.20 × 0.14 × 0.10
Crystal system	Monoclinic	Triclinic	Triclinic
Space group	$C2/c$	$P\bar{1}$	$P\bar{1}$
a (Å)	27.3395(11)	11.7722(4)	11.8395(9)
b (Å)	8.4421(4)	11.7823(4)	11.8559(8)
c (Å)	15.7903(7)	12.3804(4)	12.7320(9)
α (deg)	90	70.6953(13)	74.128(3)
β (deg)	121.7797(14)	75.3529(14)	68.833(3)
γ (deg)	90	87.6764(14)	86.828(3)
V (Å^3)	3098.1(2)	1566.24(9)	1601.2(2)
Z	8	2	2
ρ_{calcd} (g cm^{-3})	2.031	1.711	1.647
$2\vartheta_{min}/2\vartheta_{max}$ (deg)	6.418/56.094	6.922/58.038	7.072/57.324
Reflections collected/independent	16023/3729	34447/8213	30096/8018
№ of parameters/ restraints	176/0	459/0	476/0
$R1/wR2$ (all data)	0.0339/0.0756	0.0318/0.0786	0.0447/0.0868
$R1/wR2$ ($I > 2\sigma(I)$)	0.0268/0.0708	0.0276/0.0743	0.0308/0.0807
GOF	1.075	1.027	1.093
Largest diff. peak/hole (eÅ^{-3})	1.21/–0.83	0.93/–1.21	0.93/–0.82

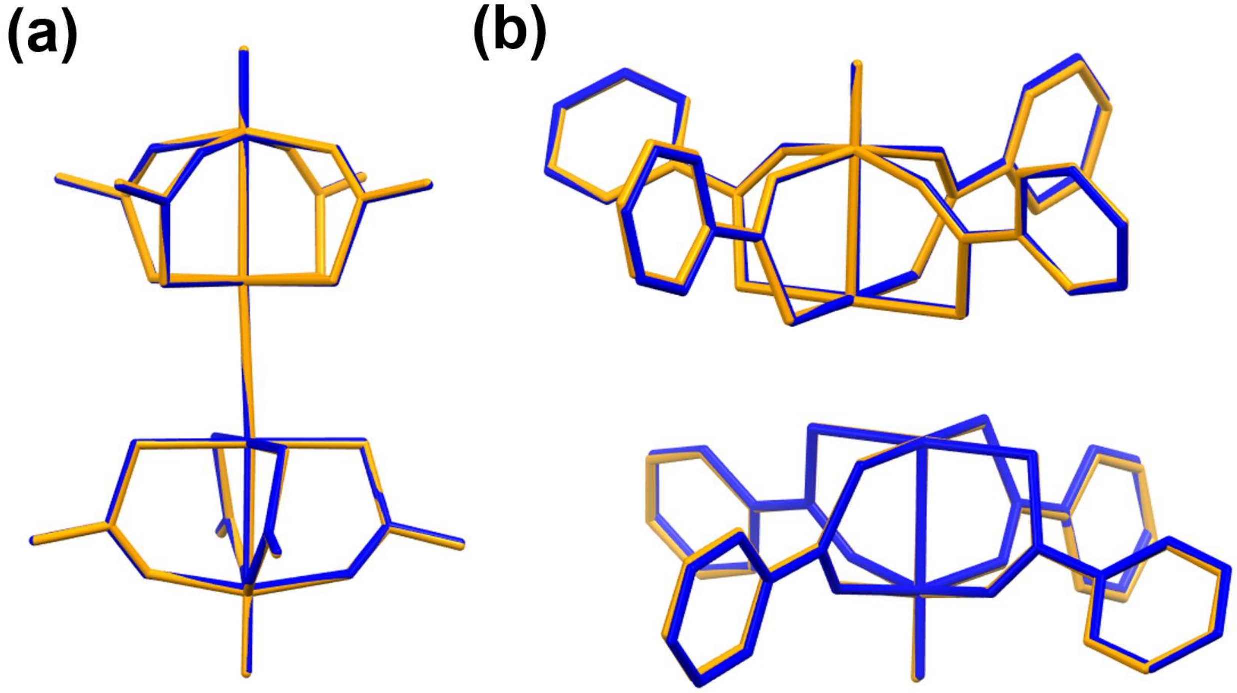

Figure S1. Overlayed structures of staggered dimers in **1Pd** and **1Pt** (a), and of square dimers in **2Pd**·DCM and **2Pt**·DCM (b). Pd- and Pt-containing paddlewheels are drawn in orange and blue, respectively. Capped sticks representation, hydrogen atoms omitted.

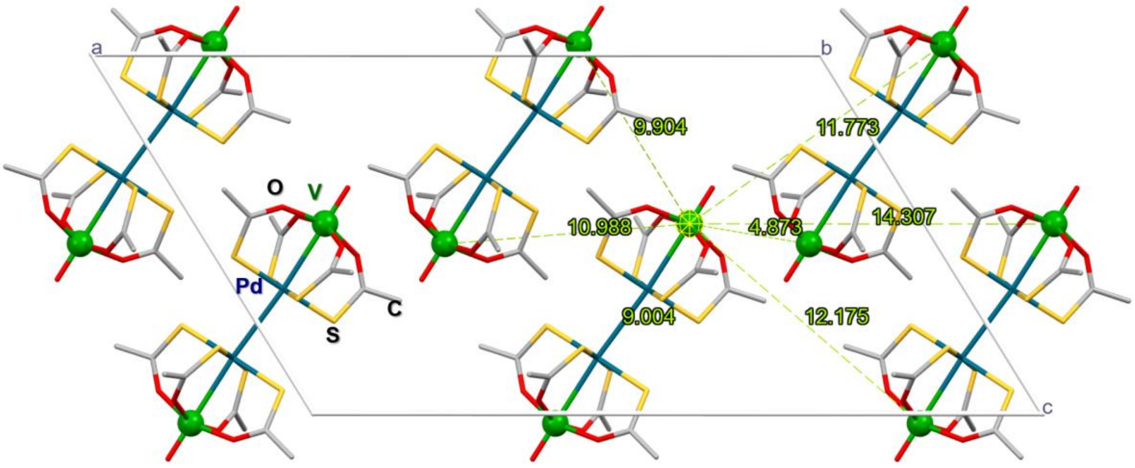

Figure S2. Shortest V···V contacts in the unit cell of **1Pd**.

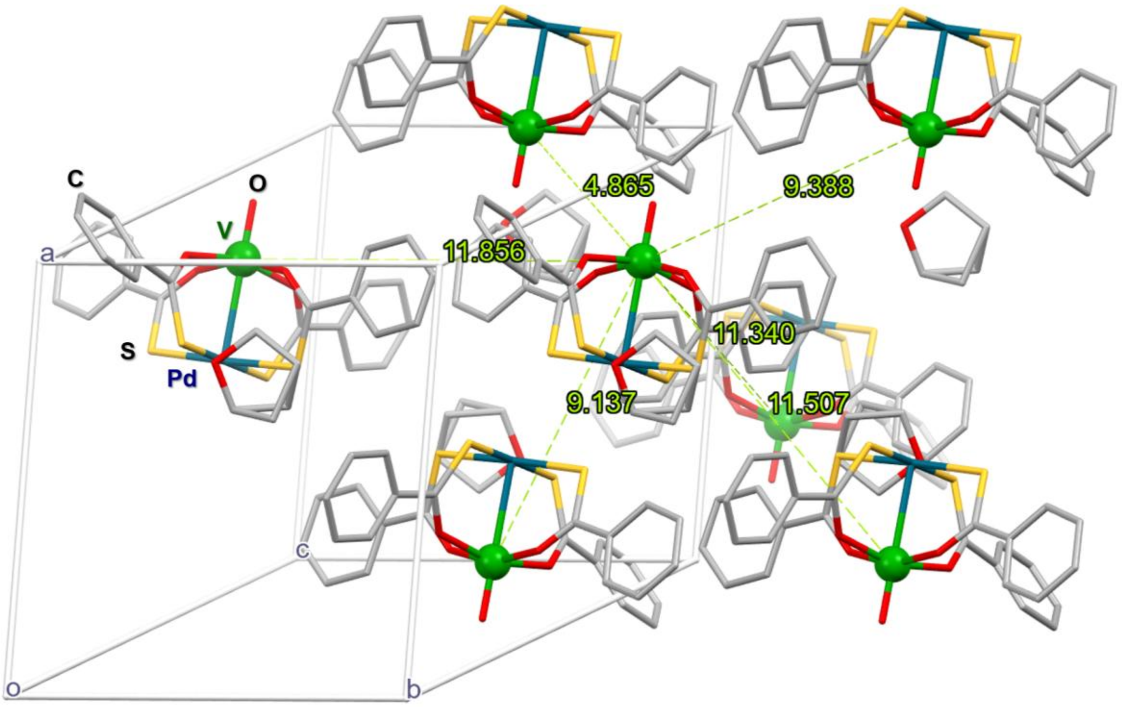

Figure S3. Shortest V···V contacts in the expanded unit cell of **2Pd**·THF.

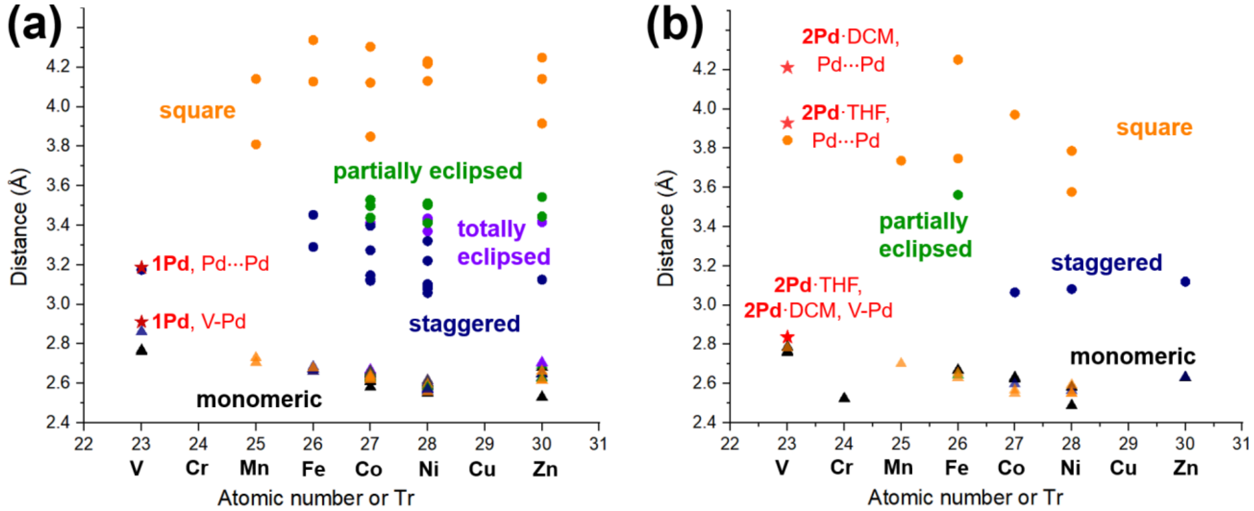

Figure S4. Statistics collected on the first-row transition metal (Tr) paddlewheels [MTr(SCOR)$_4$(L)$_n$] with R = Me (a) and Ph (b) using CCDC 2025.2.0.[1] M···M contacts displayed as circles, Tr···M as triangles. Restriction of M···M contact was set as 0–5 Å. The dimers are classified according to Doerrer's group classification.[2]

2. IR, UV-Vis, and NMR spectroscopy

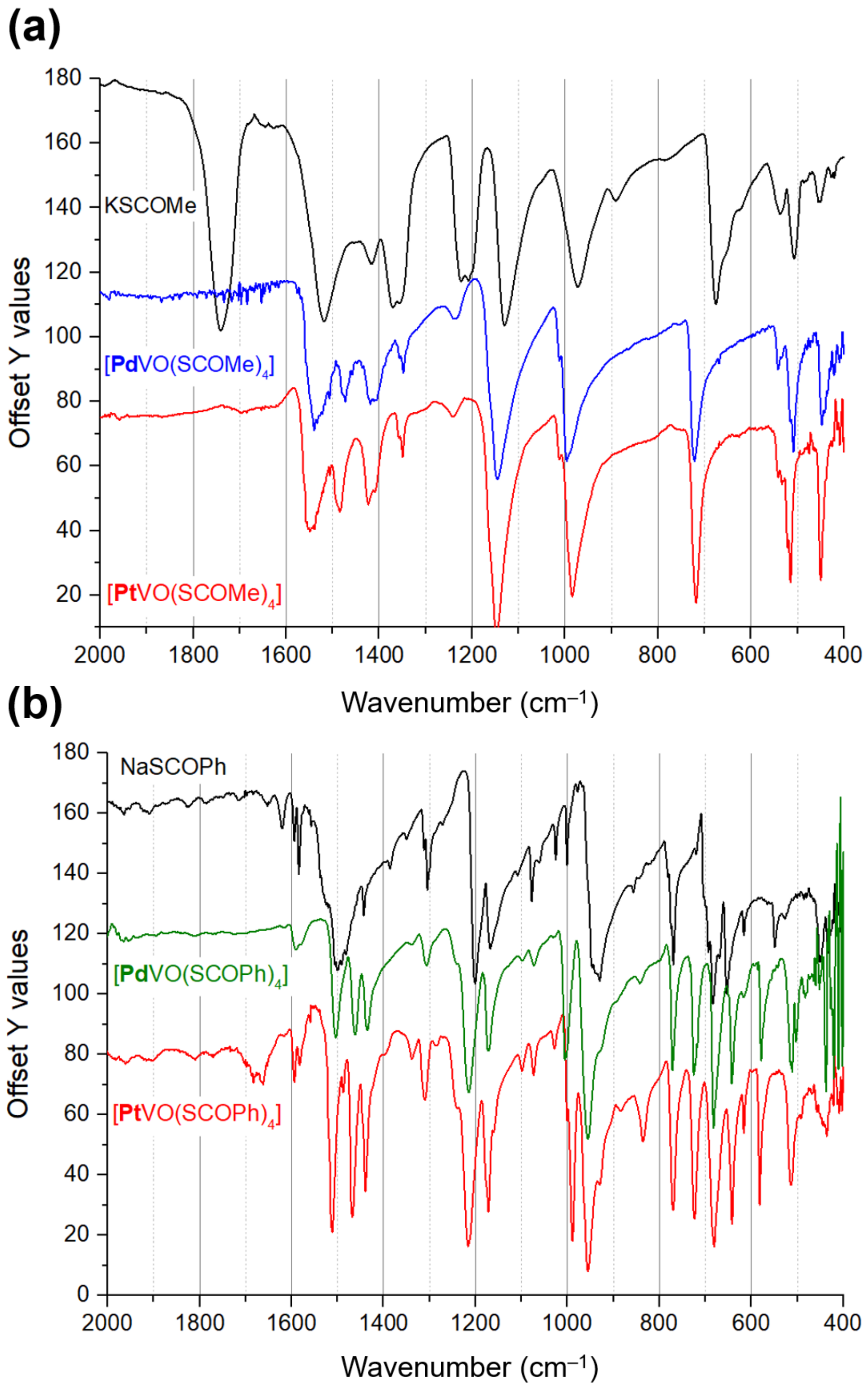

Figure S5. IR spectra of **1Pd** (a), **2Pd**·THF (b), and the corresponding alkali metals salts and Pt analogues.

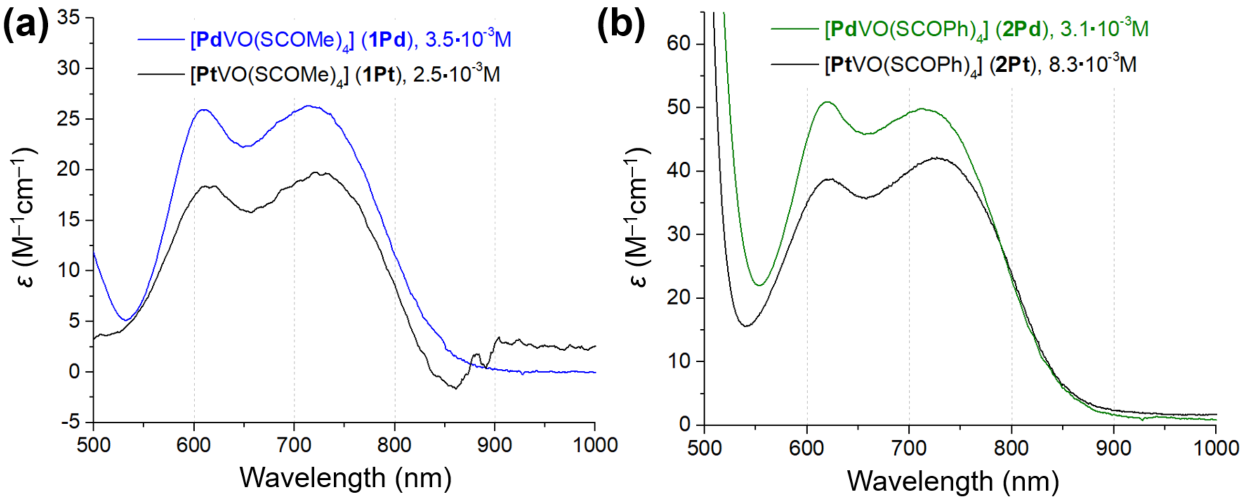

Figure S6. Electronic absorption spectra of **1Pd** (a) and **2Pd** (b) compared with the spectra of the corresponding Pt analogues in the region of vanadyl group absorption. DCM, 0.1 cm optical path length.

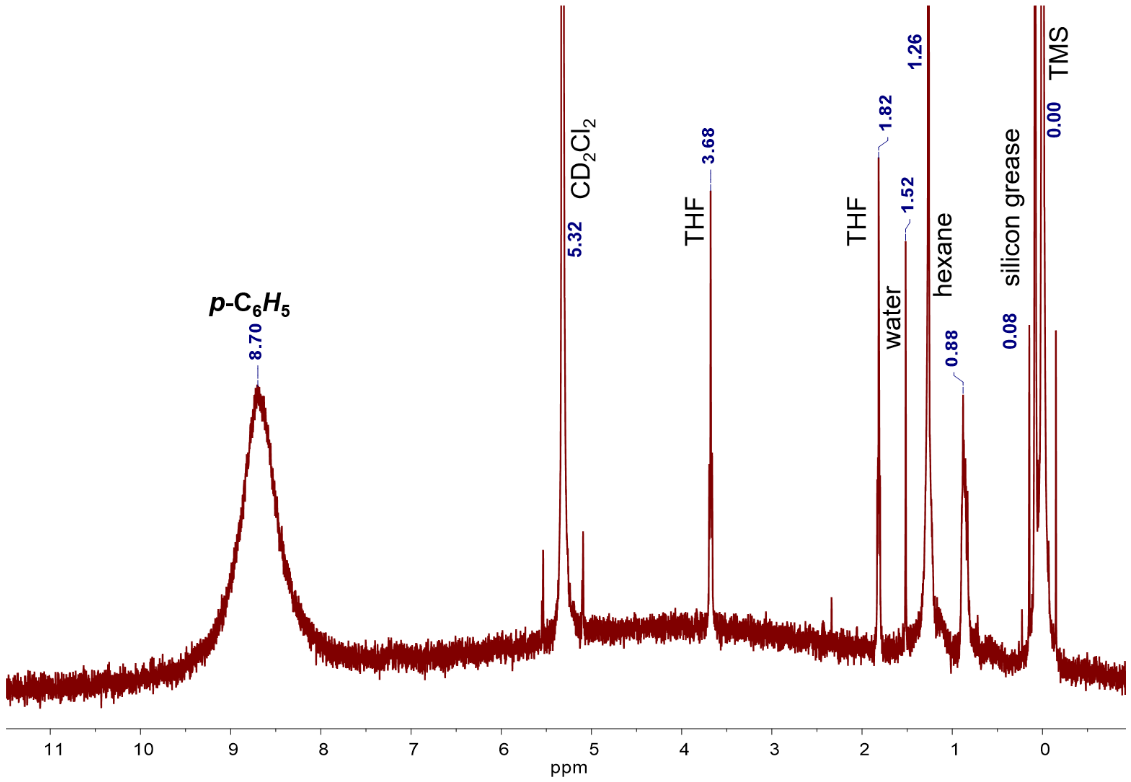

Figure S7. ^{1}H NMR spectrum of **2Pd**·THF in CD_2Cl_2. TMS was added as an internal standard for DOSY experiments. THF and *n*-hexane are residuals from crystallization and crystal washing, respectively.

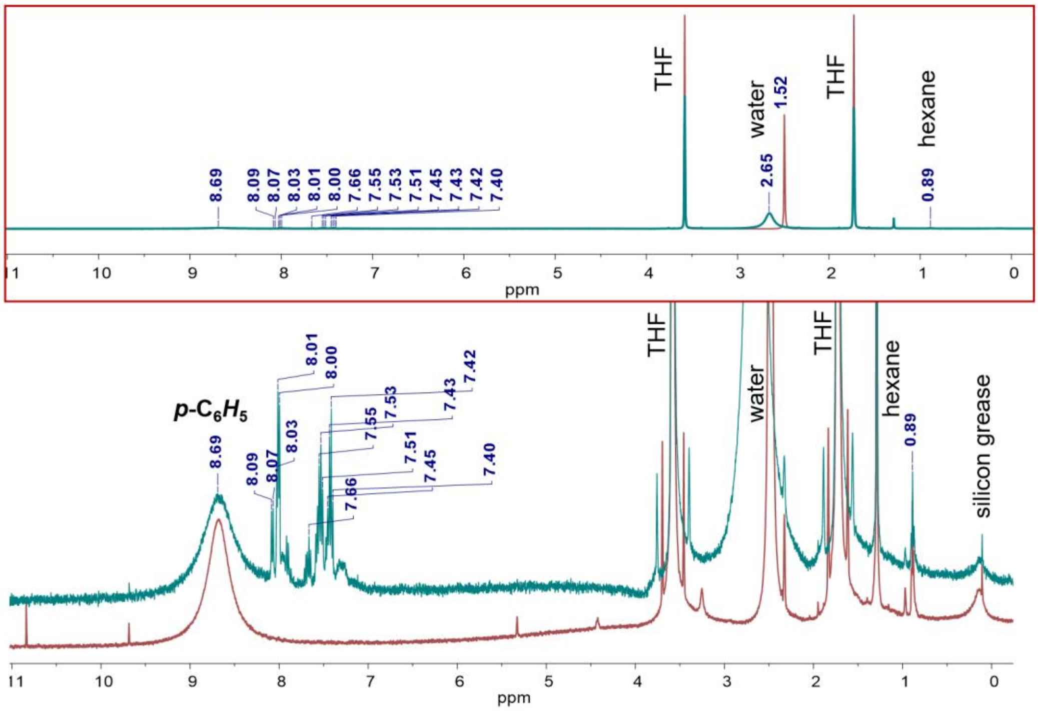

Figure S8. ^{1}H NMR spectrum of **2Pd** in wet THF-d_8, showing degradation in the presence of water. The red lower spectrum was recorded on a freshly prepared solution, and the green upper spectrum was collected 3 days later.

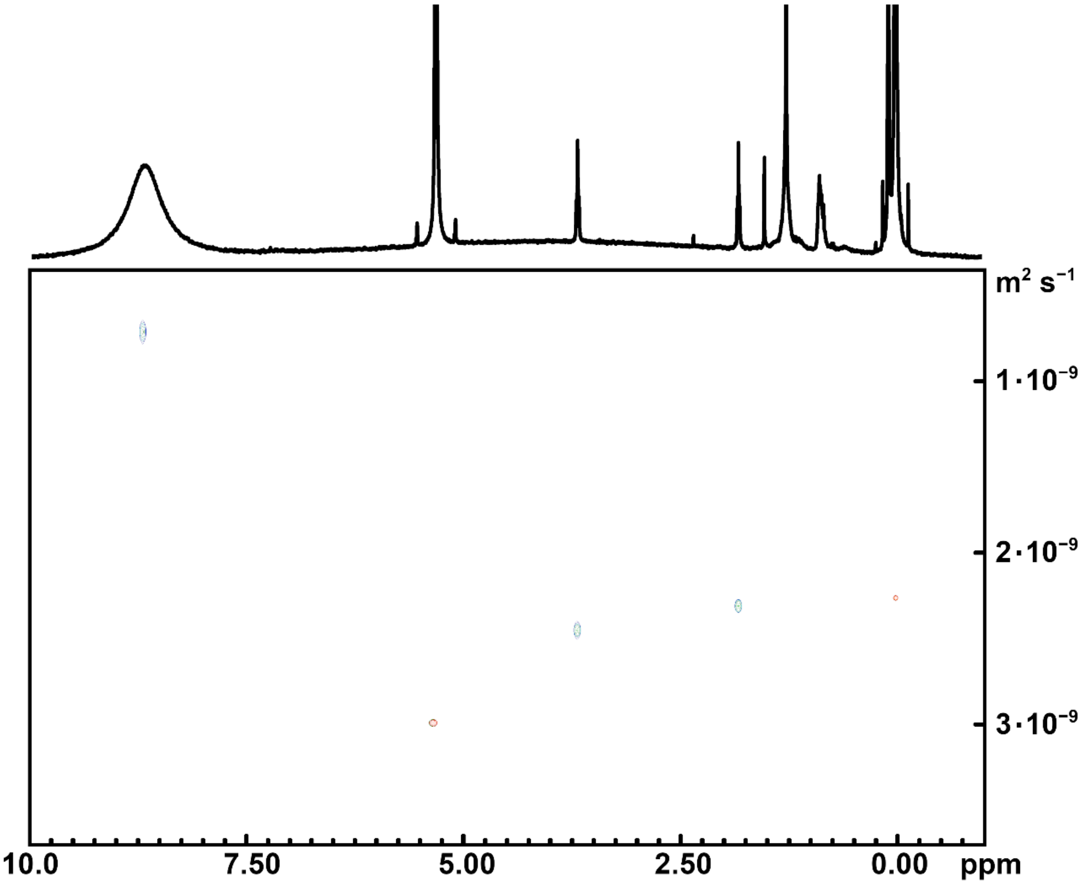

Figure S9. ^{1}H DOSY NMR spectrum of **2Pd**·THF in CD_2Cl_2 (0.01 M, 298 K, 400.13 MHz).

3. Cyclic voltammetry

Supplementary Note 1: thermodynamic parameters. Temperature-dependent electrochemical measurements showed that the $E°'$ values of both **1Pd** and **2Pd** undergo a monotonic linear decrease with increasing temperature from −30 to −5 °C (**Figure S10**). The calculated values of $\Delta S°'_{rc}$ and $\Delta H°'_{rc}$ are reported in **Table 3** together with those of the two Pt derivatives. The $E°'$ values consist of two additive contributions: a predominant enthalpic term ($-\Delta H°'_{rc}/F$) and a smaller entropic term ($T\Delta S°'_{rc}/F$), both negative in sign. The enthalpic and entropic contributions increase in the order **1Pd** < **1Pt** < **2Pt** < **2Pd** and **1Pt** < **2Pt** ~ **2Pd** < **1Pd**, respectively. It is apparent that the ordering of $E°'$ values is the same as that of the enthalpic contribution, except for **1Pt**. This derivative has a moderately negative value of $-\Delta H°'_{rc}/F$ but is the hardest to reduce because of an unusually large and unfavourable entropic contribution. Overall, the ligand dependence of $E°'$ primarily mirrors the different enthalpic contributions to the formal potential of thioacetate and thiobenzoate derivatives, while the M dependence of $E°'$ reflects a more complex interplay between enthalpic and entropic terms.

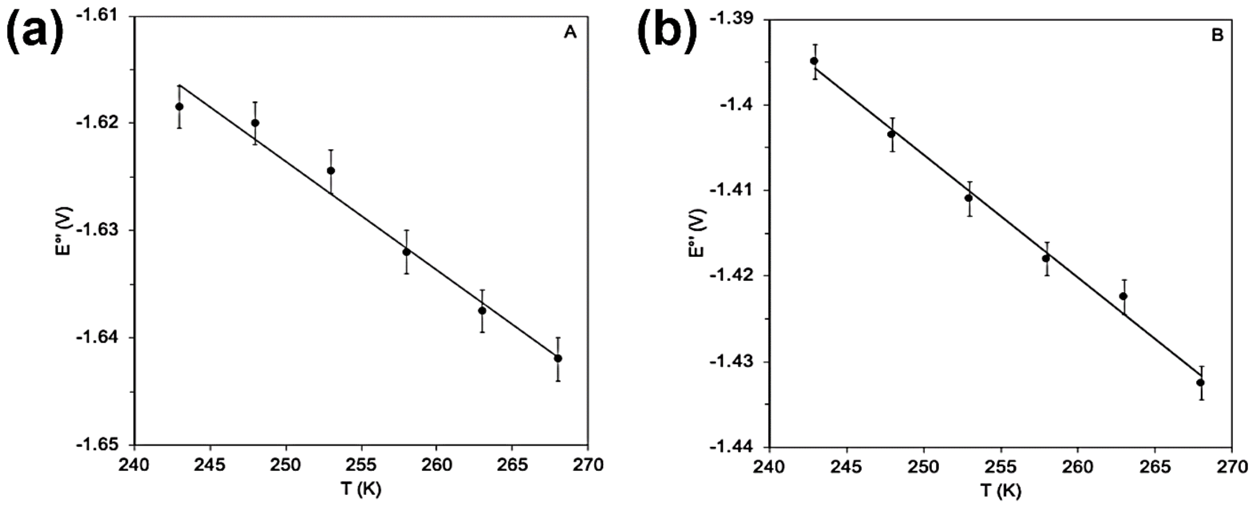

Figure S10. Plot of $E°'$ vs. T for **1Pd** (a) and **2Pd** (b) (0.5 mM solutions in DCM with 100 mM TBACl as base electrolyte).

4. EPR spectroscopy

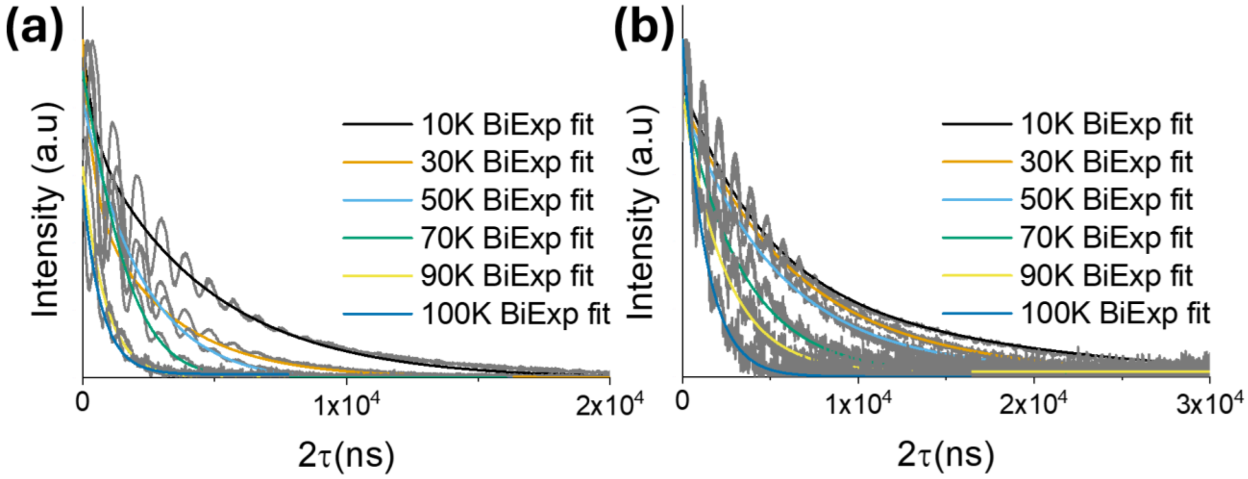

Figure S11. Echo decay traces and corresponding fits obtained using **Equation 5** for **1Pd** (a) and **2Pd** (b).

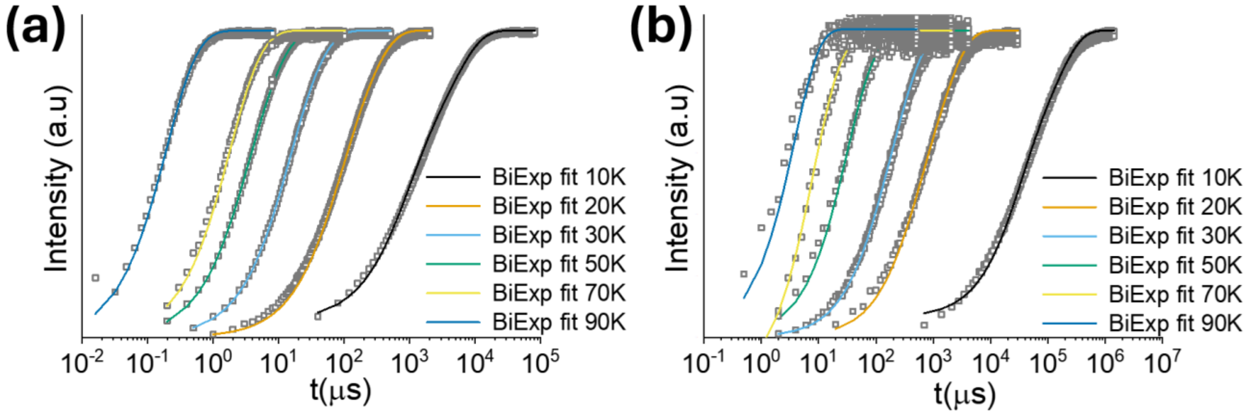

Figure S12. Inversion recovery traces and corresponding fits obtained using **Equation 4** for **1Pd** (a) and **2Pd** (b).

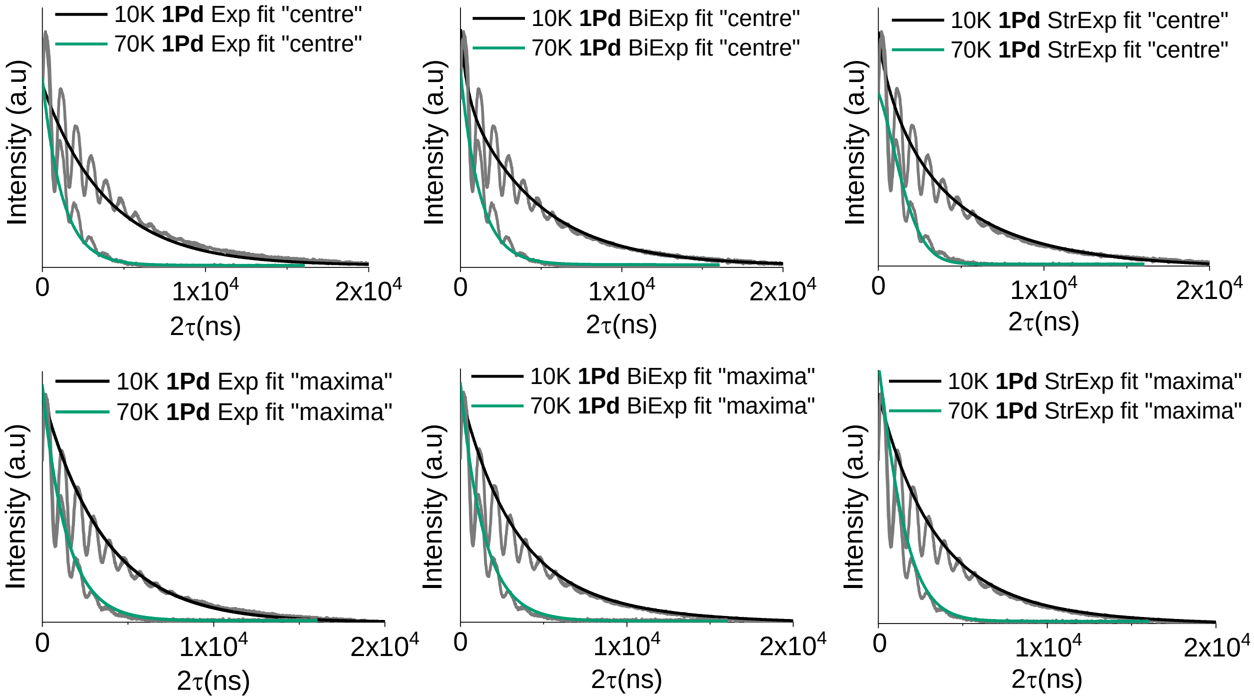

Figure S13. Different fit methods for the **1Pd** sample. Top, fit of the center of the ESEEM modulation using from left to right **Equations 6** (mono-exponential decay), **5** (bi-exponential decay), and **7** (stretched-exponential decay); bottom fit of the maxima of the ESEEM modulations using the same models. The corresponding T_m values extracted from the different methods are listed in **Table S3**.

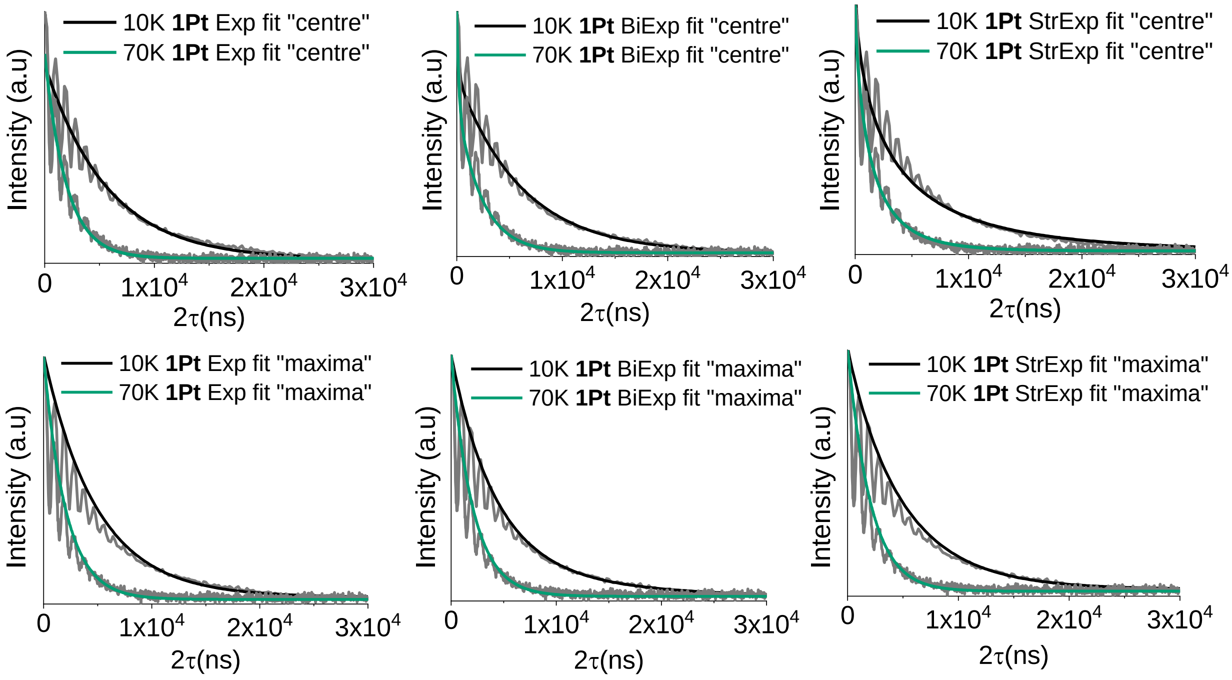

Figure S14. Different fit methods for the **1Pt** sample. The data are taken from Ref. [3]. Top, fit of the center of the ESEEM modulation using from left to right **Equations 6** (mono-exponential decay), **5** (bi-exponential decay), and **7** (stretched-exponential decay); bottom fit of the maxima of the ESEEM modulations using the same models. The corresponding T_m values extracted from the different methods are listed in **Table S3**.

Table S2. T_1 and T_m values extracted from the biexponential fit of the experimental inversion recovery and echo decay traces (**Equations 4** and **5**). The fits are presented in **Figures S11** and **S12**.

Compound	T (K)	T_1 (μs)				T_m (μs)			
		A_{slow}	T_1 slow	A_{fast}	T_1 fast	A_{slow}	T_m slow	A_{fast}	T_m fast
**1Pd**	10	52	4.5×10^3	48	8.0×10^2	75	4.5	25	0.38
	20	51	9.3×10^2	49	219	65	3.6	35	0.44
	30	55	2.1×10^2	45	62	59	3.0	41	0.45
	50	52	26	48	11	-	2.3	-	2.3
	70	50	6.7	50	2.7	-	1.7	-	1.7
	90	21	3.6	79	1.6	-	0.74	-	0.74
	100	84	2.0	16	1.0	-	0.70	-	0.70
**2Pd**	10	51	1.4×10^4	49	2.5×10^3	61	12	39	3.0
	20	65	1.6×10^3	35	3.7×10^2	85	9.0	15	1.5
	30	68	3.0×10^2	32	89	-	5.2	-	5.2
	50	36	60	64	25	-	2.5	-	2.5
	70	47	14	53	5.9	-	2.3	-	2.3
	90	65	4.8	35	1.8	-	1.3	-	1.3
	100	-	2.5	-	2.5	-	0.565	-	0.565

Table S3. Best fit parameters for **1Pd** and **1Pt** using different fitting functions (**Equations 5-7**). The fitting curves are shown in **Figures S13** and **S14**.

Compound	T (K)	T_m (μs)							
		monoexponential	biexponential					stretched exponential	
			A_{slow}	$T_{m,slow}$	A_{fast}	$T_{m,fast}$	$T_{m,av}$	$T_{m,str}$	β
Fit considering the "center" of the ESEEM oscillations									
**1Pd**	10	4.0	74	4.5	26	0.38	3.4	3.4	0.8
	70	1.7	-	1.7	-	1.7	1.7	2.0	1.7
**1Pt**	10	5.7	72	6.1	28	0.169	4.4	3.1	0.7
	70	2.0	62	2.3	38	0.182	1.5	1.4	0.7
Fit considering the maxima of the ESEEM oscillations									
**1Pd**	10	3.6	20	7.0	80	3.0	3.8	3.5	0.9
	70	1.4	-	1.4	-	1.4	1.4	1.6	1.2
**1Pt**	10	5.0	11	12.6	89	4.3	5.2	4.8	1.0
	70	2.0	-	2.0	-	2.0	2.0	2.0	1.0

Table S4. Best fit parameters in **Equation 1** for **1Pd** and **2Pd**.

Compound	A_{dir} [Hz]	A_{loc} [MHz]	$\hbar\omega_{loc}$ [cm^{-1}]
**1Pd**	12	1.5	122
**2Pd**	3.62	0.62	107

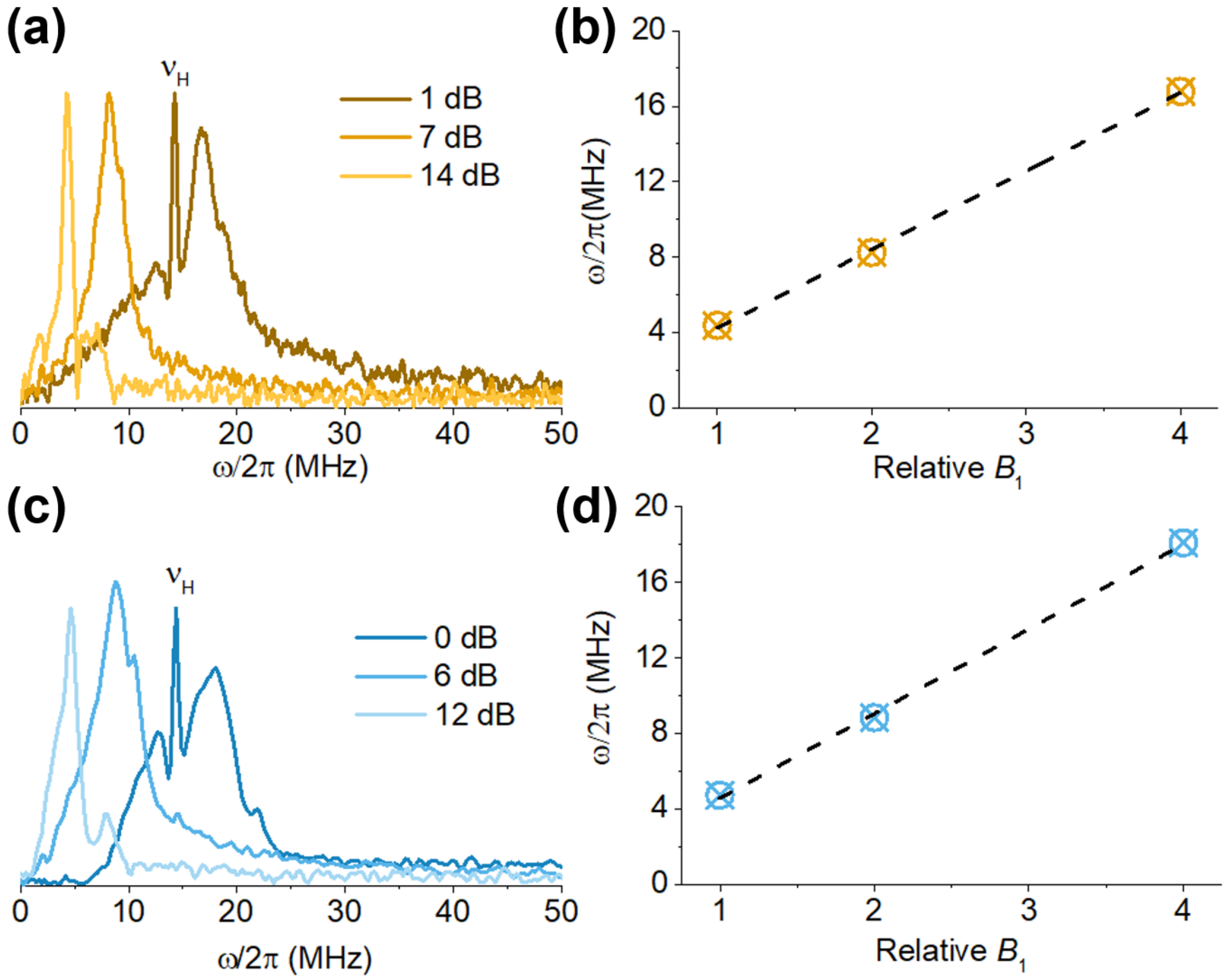

Figure S15. Rabi frequency as a function of the relative intensity of the oscillating field B_1 for **1Pd** (yellow) and **2Pd** (blue).

Supplementary Note 2: simulation analysis of ^{105}Pd hyperfine coupling. In our previous study on **1Pt** and **2Pt**,[3] clear hyperfine splittings of the ^{51}V EPR signals were observed, arising from the interaction of the unpaired electron with the ^{195}Pt nucleus (I = 1/2, 33.83% natural abundance, g_n^{Pt} = 1.2190). From the measured hyperfine tensors — [in **1Pt**: $^{Pt}A_z$ = 80.0(5) MHz, $^{Pt}A_{x,y}$ = 47(2) MHz; in **2Pt**: $^{Pt}A_z$ = 74.0(5) MHz, $^{Pt}A_{x,y}$ = 42(2) MHz] — a spin density transfer to the Pt atoms of approximately ρ_{Pt} = 2.7% was determined for both complexes.

In contrast, the CW-EPR spectra of the Pd analogues **1Pd** and **2Pd** show no detectable hyperfine structure attributable to the magnetically active ^{105}Pd isotope (I = 5/2, 22.33% natural

abundance, g_n^{Pd} = –0.2570). Assuming the same spin density transfer as in the Pt complexes, the expected ^{105}Pd hyperfine coupling constants can be estimated by scaling the elements of the $^{Pt}\bar{\bar{\mathbf{A}}}$ tensor according to the ratio of the nuclear g-factors. Applying the described approach to the thioacetate derivatives **1Pt** and **1Pd**, one obtains:

$$[{}^{Pd}A_x, {}^{Pd}A_y, {}^{Pd}A_z] = [{}^{Pt}A_x, {}^{Pt}A_y, {}^{Pt}A_z] \times \left|\frac{g_n^{Pd}}{g_n^{Pt}}\right| = [47, 47, 80] \times \left|\frac{-0.2570}{1.2190}\right| = [9.9, 9.9, 16.9]\ \mathrm{MHz}$$

The corresponding spectral simulation, performed under this assumption, is shown in **Figure S16**. The simulated spectrum (red trace) displays clear ^{105}Pd hyperfine satellites, which are not observed in the experimental data (**Figure S16b**).

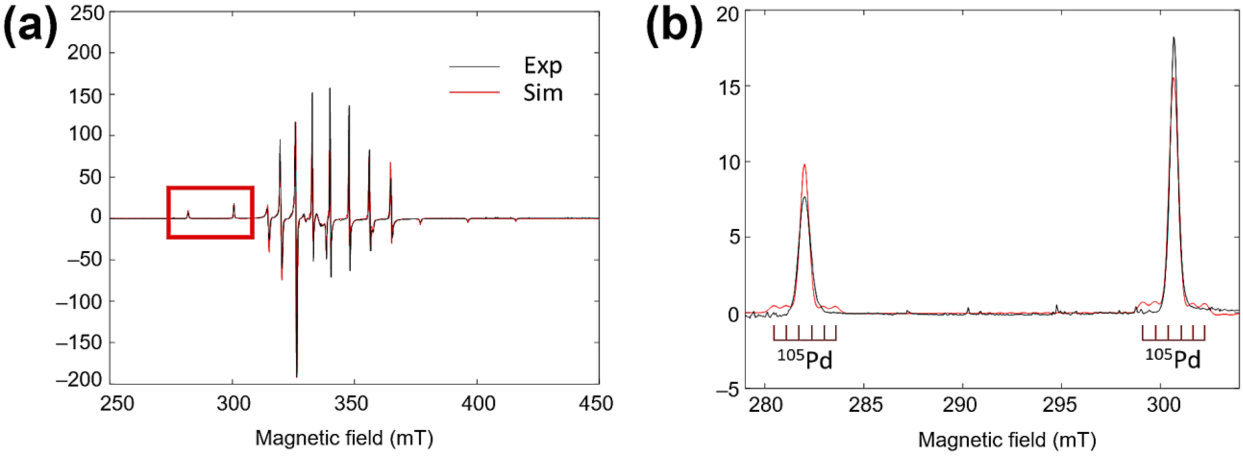

Figure S16. Computer simulation of the X-band CW-EPR spectrum of **1Pd** based on the assumption that the spin density transfer on Pd is the same as that observed in the Pt analogue (see text). The figure shows both the full spectrum (a) and a magnification (b) of the low field transitions (^{51}V: m_I = –7/2 and m_I = –5/2, red square in **Figure S16a**). The following parameters were used in the simulation: ν = 9.457776 GHz, $\bar{\bar{\mathbf{g}}}$ = [1.982, 1.982, 1.935], $^{V}\bar{\bar{\mathbf{A}}}$ = [197, 197, 518] MHz, $^{Pd}\bar{\bar{\mathbf{A}}}$ = [9.9, 9.9, 16.9] MHz. A Gaussian line shape with full width at half maximum (FWHM) of 0.5 mT was employed. This ensures that broadening effects are not related to the low abundance ^{105}Pd nuclei. In **Figure S16b**, the stick diagram highlights the ^{105}Pd hyperfine transitions

To further investigate the extent of ^{105}Pd hyperfine coupling, we performed a series of spectral simulations starting from the DFT-computed hyperfine tensor for **1Pd**, namely $^{Pd}\bar{\bar{\mathbf{A}}}$ = [2.6, 2.6, 5.3] MHz. The results are presented in **Figure S17**. When no hyperfine interaction with ^{105}Pd is included (**Figure S17a**), the simulated spectral linewidth does not match the experimental lineshape. The best agreement is achieved using the DFT-calculated tensor (**Figure S17b**), whereas larger coupling values lead to excessive broadening in the spectral wings. Based on this analysis, a maximum ^{105}Pd hyperfine coupling of approximately 5 MHz can be inferred, which implies a reduced spin density transfer with respect to the Pt complex.

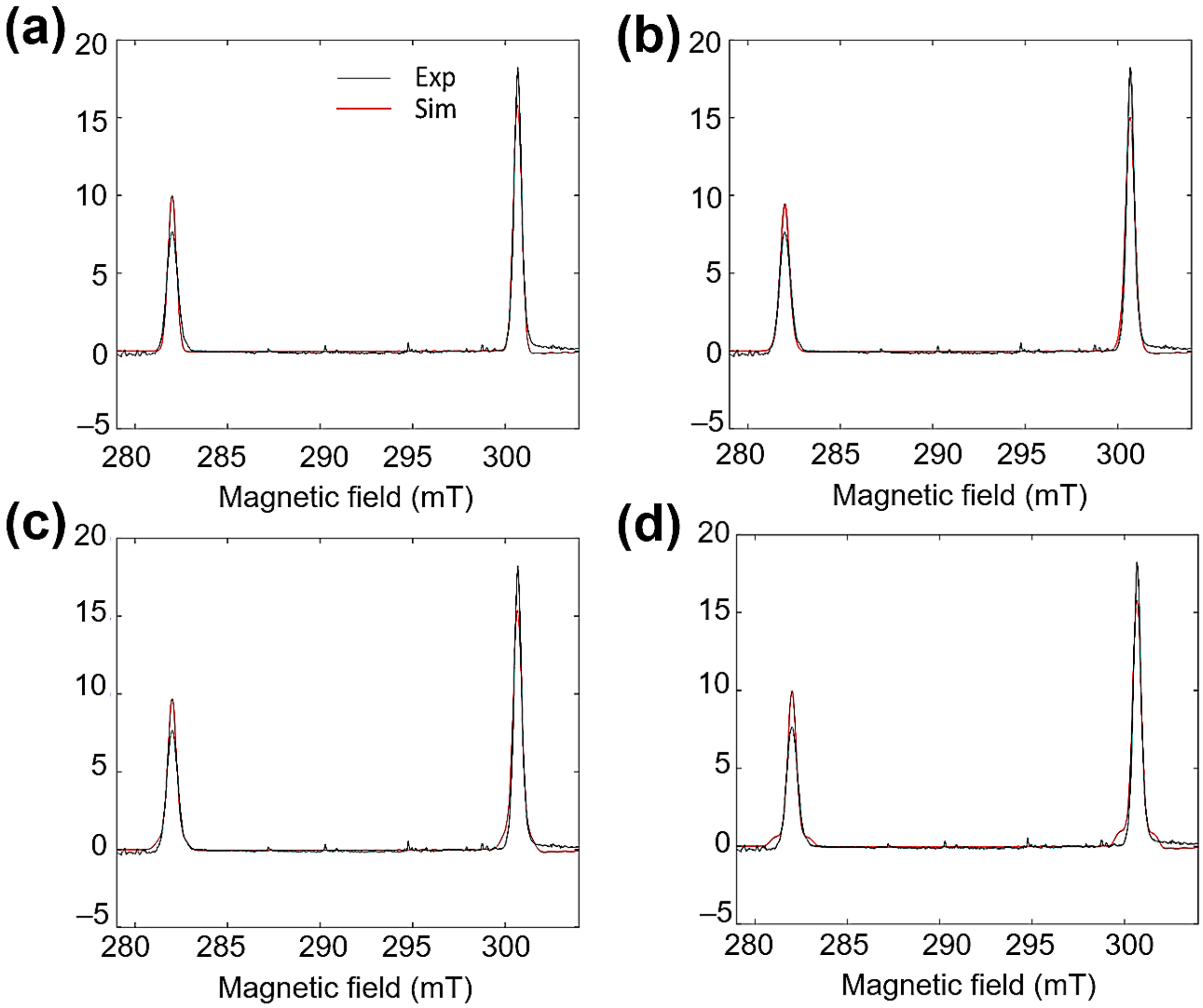

Figure S17. Computer simulation of the X-band CW-EPR spectrum of **1Pd** assuming different ^{105}Pd hyperfine tensors. Only the low field transitions (^{51}V: m_I = –7/2 and m_I = –5/2) are shown. (a) $^{Pd}\bar{\bar{\mathbf{A}}}$ = 0 MHz, (b) $^{Pd}\bar{\bar{\mathbf{A}}}$ = [2.6, 2.6, 5.3] MHz, (c) $^{Pd}\bar{\bar{\mathbf{A}}}$ = [2.6, 2.6, 5.3]x1.5 = [3.9, 3.9, 7.95] MHz, (d) $^{Pd}\bar{\bar{\mathbf{A}}}$ = [2.6, 2.6, 5.3]x2 = [5.2, 5.2, 10.6] MHz. All other parameters are the same as those reported in the caption of **Figure S16.**

5. DFT calculations

Table S5. Contributions to the ^{105}Pd hyperfine coupling tensor for **1Pd** and **2Pd** as computed by DFT. All values are in MHz.

**1Pd**	$^{Pd}A_x$	$^{Pd}A_y$	$^{Pd}A_z$	*Iso*
Fermi contact	3.3302	3.3302	3.3302	3.3302
spin-dipole	–0.4582	–0.4595	0.9177	-
spin-orbit	–0.2709	–0.2701	1.0900	0.1830
Total	2.6011	2.6005	5.3378	3.5131

**2Pd**	$^{Pd}A_x$	$^{Pd}A_y$	$^{Pd}A_z$	*Iso*
Fermi contact	3.2554	3.2554	3.2554	3.2554
spin-dipole	–0.4135	–0.4149	0.8284	-
spin-orbit	–0.2487	–0.2481	1.0002	0.1678
Total	2.5932	2.5924	5.0839	3.4231

Table S6. Calculated vibrational frequencies below 200 cm^{-1} for **1Pd** and **1Pt**.

Normal Mode	Frequency (cm^{-1})	
	**1Pd**	**1Pt**
6	53.88	60.82
7	58.17	61.28
8	58.90	62.04
9	59.27	69.08
10	93.48	103.65
11	94.71	107.87
12	95.51	108.15
13	104.07	112.45
14	106.17	116.42
15	107.29	119.92
16	116.98	124.84
17	125.25	128.02
18	146.30	140.60
19	162.39	164.95
20	164.07	168.43
21	164.67	170.69
22	187.44	184.71
23	188.34	188.33
24	191.58	198.18
25	199.24	-

Table S7. Calculated vibrational frequencies below 200 cm^{-1} for **2Pd** and **2Pt**.

Normal Mode	Frequency (cm^{-1})	
	**2Pd**	**2Pt**
6	17.48	7.64
7	19.10	8.03
8	19.45	11.97
9	27.90	14.80
10	29.11	15.34

11	29.74	30.15
12	33.27	31.93
13	40.49	35.72
14	46.92	43.75
15	67.44	68.83
16	77.31	77.41
17	77.81	80.97
18	83.28	96.08
19	109.74	106.46
20	109.91	117.70
21	112.78	124.44
22	124.04	125.44
23	124.31	128.22
24	127.32	131.45
25	137.24	135.07
26	150.45	155.89
27	176.28	168.43
28	176.50	170.61
29	176.86	171.92
30	185.40	175.07
31	187.31	194.46
32	-	196.90

6. References

(1) Groom, C. R.; Bruno, I. J.; Lightfoot, M. P.; Ward, S. C. The Cambridge Structural Database. *Acta Crystallogr. B. Struct. Sci. Cryst. Eng. Mater.* **2016**, *B72* (2), 171–179. https://doi.org/10.1107/S2052520616003954.

(2) Beach, S. A.; Doerrer, L. H. Heterobimetallic Lantern Complexes and Their Novel Structural and Magnetic Properties. *Acc. Chem. Res.* **2018**, *51* (5), 1063–1072. https://doi.org/10.1021/acs.accounts.7b00585.

(3) Imperato, M.; Nicolini, A.; Borsari, M.; Briganti, M.; Chiesa, M.; Liao, Y. K.; Ranieri, A.; Raza, A.; Salvadori, E.; Sorace, L.; Cornia, A. Quantum Spin Coherence and Electron Spin Distribution Channels in Vanadyl-Containing Lantern Complexes. *Inorg. Chem. Front.* **2024**, *11*, 186–195. https://doi.org/10.1039/d3qi01806g.
